# Current Perspectives on STING Agonists for Anticancer Drug Development

**DOI:** 10.1111/cbdd.70370

**Published:** 2026-07-26

**Authors:** Dilay Kahvecioglu Cicek

**Affiliations:** ^1^ Edirne Sultan 1. Murat State Hospital, Republic of Türkiye Ministry of Health Edirne Türkiye

**Keywords:** cancer immunotherapy, cyclic dinucleotide (CDN) agonist, cyclic GMP‐AMP synthase (cGAS)—stimulator of interferon genes (STING) pathway, human STING, non‐cyclic dinucleotide (non‐CDN) agonist, STING agonist, STING pathway

## Abstract

The cyclic GMP‐AMP (cGAS) synthase and stimulator of interferon genes signaling pathway plays a central role in bridging innate and adaptive immunity, particularly within the context of cancer. Activation begins when cytosolic double‐stranded DNA is detected, leading to the production of cyclic GMP‐AMP (cGAMP) and subsequent activation of STING. This initiates a cascade that induces the expression of type I interferons (IFN) and proinflammatory cytokines, enhancing antitumor immune responses through the stimulation of dendritic cells, cytotoxic T lymphocytes, and natural killer cells. Despite strong therapeutic potential, many currently available cyclic dinucleotide (CDN) and synthetic noncyclic dinucleotide (non‐CDN) STING agonists face critical limitations. Common issues include poor pharmacokinetics, low cellular permeability, enzymatic degradation, and inadequate systemic bioavailability. In some cases, excessive immune activation has been observed, resulting in toxicity, chronic inflammation, or immunosuppressive tumor microenvironments. Furthermore, species‐specific activity restricts the translational relevance of several compounds. These limitations highlight the need for the development of novel STING agonists with improved potency, selectivity, safety, and pharmacological profiles. This review presents a detailed analysis of molecular design approaches and structure–activity relationship (SAR) data for STING agonists, emphasizing their relevance in cancer therapy. A total of 60 synthetic compounds with diverse chemical scaffolds are examined to identify structural features linked to enhanced STING activation. These findings may support the discovery of novel STING‐targeted molecules that could improve therapeutic outcomes in cancer treatment.

## Graphical abstract

The molecular structures of STING agonists play a decisive role in regulating the STING pathway. Their distinct scaffolds and functional groups orchestrate receptor binding, conformational activation, and the subsequent immune response.

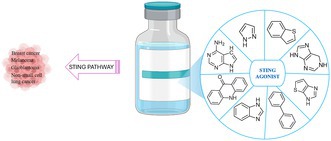

## 1 Introduction

The activation of the cyclic GMP‐AMP synthase (cGAS) and stimulator of interferon genes (STING) pathway plays a pivotal role in bridging innate and adaptive immunity, especially in the context of cancer. This signaling cascade is initiated when cytoplasmic double‐stranded DNA (dsDNA) is detected, which can occur as a result of DNA damage, viral infections, mitochondrial stress, or genomic instability (Shen et al. 2025). Under normal conditions, mammalian DNA is confined to the nucleus or mitochondria, and very little is present in the cytoplasm. However, when cellular stress or damage leads to the presence of dsDNA in the cytosol, cGAS recognizes this abnormal DNA in a sequence‐independent manner and catalyzes the synthesis of cyclic GMP‐AMP (cGAMP) from adenosine triphosphate (ATP) and guanosine triphosphate (GTP).

**FIGURE 1:**
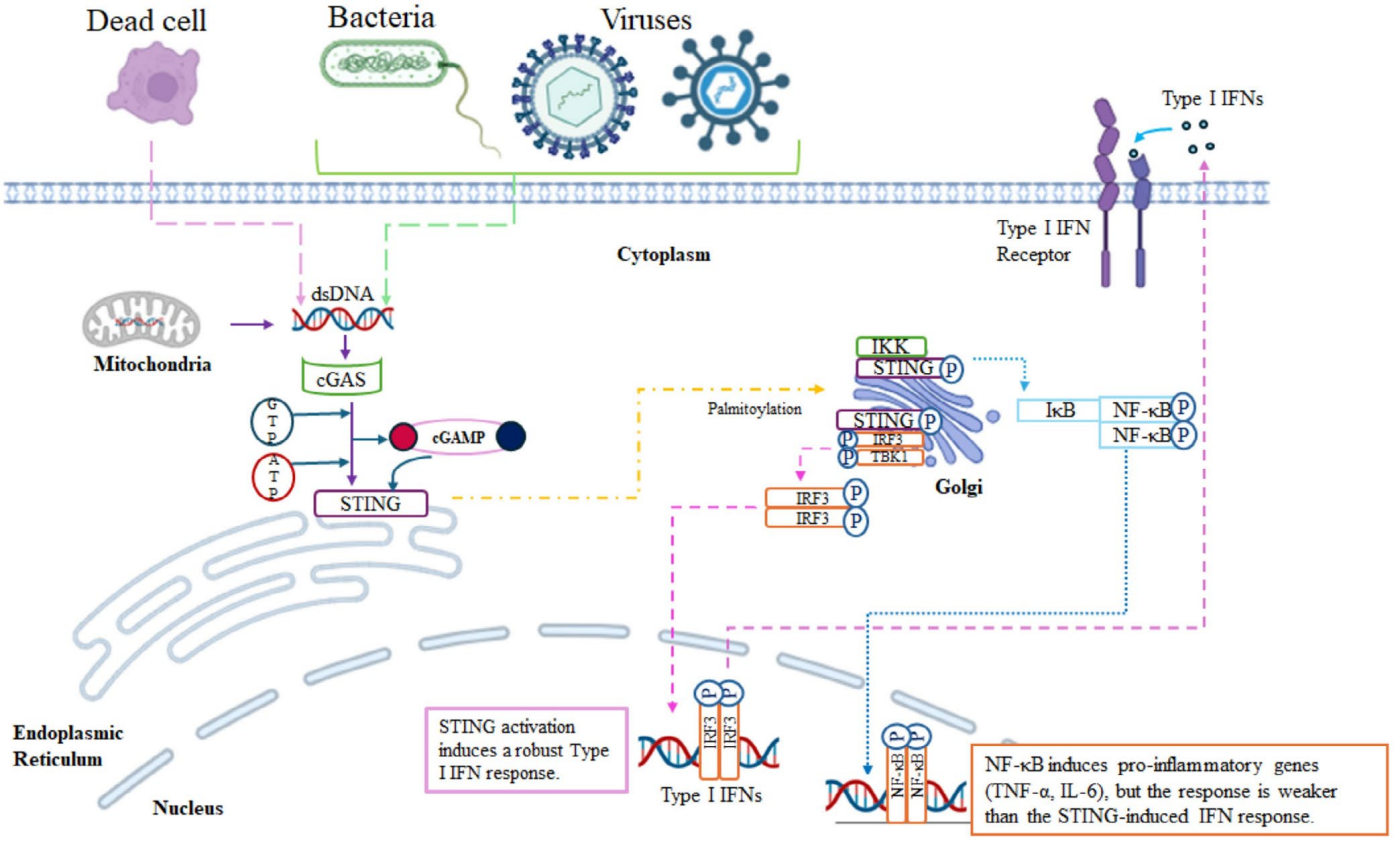
Schematic illustration of the cGAS‐STING signaling pathway. Upon recognition of cytosolic dsDNA (from viruses, bacteria, or mitochondria), cGAS catalyzes the synthesis of the second messenger cGAMP from ATP and GTP. cGAMP binds to STING dimers at the ER, triggering their translocation to the Golgi apparatus. At the Golgi, STING undergoes palmitoylation at Cys88/91, a critical step that facilitates the recruitment of TBK1. Subsequently, TBK1 phosphorylates STING and IRF3. Phosphorylated IRF3 translocates to the nucleus to drive the expression of IFNs. The pathway also activates NF‐κB, inducing the production of proinflammatory cytokines (e.g., TNF‐α, IL‐6).

cGAMP functions as a second messenger by binding to STING, an adaptor protein primarily located on the endoplasmic reticulum (ER). Upon binding, STING undergoes a conformational change, oligomerizes, and translocates to the Golgi apparatus. Palmitoylation is an essential posttranslational modification required for STING activation after translocation from the ER to the Golgi. STING becomes palmitoylated at the membrane‐proximal cysteine residues Cys88 and Cys91, as shown in Figure 1. This modification is indispensable for initiating downstream signaling; without it, STING cannot activate TBK1 and IRF3, failing to induce type I interferons or other STING‐dependent immune genes. Consequently, inhibition of palmitoylation (e.g., by 2‐bromopalmitate) or mutation of Cys88/91 abolishes the type I interferon (IFN‐I) response. Thus, while not required for ER‐to‐Golgi trafficking, palmitoylation at the Golgi acts as a key regulatory switch enabling full STING activation (Mukai et al. 2016). At the Golgi, palmitoylation further facilitates the efficient recruitment and activation of downstream signaling molecules, particularly TANK‐binding kinase 1 (TBK1) and IκB kinase (IKK) (Nicolai et al. 2020; Xie et al. 2020). These kinases phosphorylate the transcription factors interferon regulatory factor 3 (IRF3) and nuclear factor kappa‐light‐chain‐enhancer of activated B cells (NF‐κB), which then translocate to the nucleus and induce the transcription of IFN‐I and proinflammatory cytokines such as interleukin‐6 (IL‐6), CXCL10, and tumor necrosis factor‐alpha (TNF‐α) (Gehrcken et al. 2025).

The immune mediators produced through cGAS‐STING signaling stimulate a robust antitumor immune response (Podojil et al. 2022). They promote the maturation and antigen‐presenting capabilities of dendritic cells (DCs), enhance cross‐priming of CD8^+^ cytotoxic T lymphocytes, and activate natural killer (NK) cells (Li et al. 2020; Nicolai et al. 2020). These effects contribute to the immune system's detection and elimination of tumor cells. In addition, interferons increase the expression of major histocompatibility complex class I (MHC‐I) molecules and extend antigen presentation duration by inhibiting endo‐lysosomal acidification in DCs, thereby boosting immune recognition of tumor antigens.

The activation of STING also influences NK cell function by promoting the production of interleukin‐15 (IL‐15) in the tumor microenvironment, which leads to the expansion of IL‐15 receptor‐positive NK cells with enhanced cytotoxic capacity. Furthermore, the tumor‐derived messenger cGAMP can stimulate the expression of transcription factor T‐cell factor 1 (TCF‐1) in NK cells, contributing to the generation of memory‐like features and prolonged antitumor responses (Lu et al. 2023).

Because of its strong ability to activate the immune system, the cGAS STING pathway is considered a promising target in cancer immunotherapy. Researchers are developing pharmacological STING agonists to enhance immune responses in tumors that usually avoid detection. In addition, combining STING agonists with immune checkpoint inhibitors such as programmed cell death protein 1 (PD‐1) antibodies including pembrolizumab and spartalizumab or programmed cell death ligand 1 (PD L1) antibodies has shown potential to overcome resistance to treatment and improve overall effectiveness. Despite its significant antitumor effects, the cGAS‐STING pathway also exhibits protumorigenic properties under certain conditions (Xue et al. 2022). Chronic or excessive activation of STING may lead to persistent inflammation and the creation of an immunosuppressive tumor microenvironment. This environment is characterized by the recruitment of regulatory T cells (Tregs), myeloid‐derived suppressor cells, and the upregulation of immune checkpoint molecules such as indoleamine 2,3‐dioxygenase (Zhang et al. 2024). These changes suppress CD8^+^ T cell infiltration and cytotoxic function, facilitating immune escape and tumor progression.

While reduced STING expression is generally linked to immune evasion, paradoxical findings suggest that chronic, noncanonical activation of the cGAS‐STING pathway can also correlate with poor prognosis in certain gastrointestinal cancers, such as colorectal and gastric adenocarcinomas. This detrimental effect is often attributed to sustained inflammatory signaling or the activation of noncanonical NF‐κB pathways, which can promote tumor invasion and metastasis (Yao et al. 2022). Additionally, chromosomally unstable tumor cells may exploit continuous STING activation to desensitize the immune response or promote an immunosuppressive microenvironment, further supporting immune evasion (Beernaert and Parkes 2023). Thus, the clinical impact of STING appears context‐dependent, necessitating a careful evaluation of its expression levels and signaling intensity (Ruiz‐Iglesias et al. 2025).

In conclusion, the cGAS‐STING pathway is a central regulator of innate immunity with profound implications for cancer biology. It can enhance antitumor immunity by activating interferon responses and stimulating cytotoxic immune cells, but it can also promote tumor growth when activated persistently or inappropriately. Understanding the balance between these opposing effects is essential for the development of safe and effective therapies targeting this pathway. Continued research into STING agonists will help refine strategies for integrating cGAS‐STING modulation into cancer immunotherapy.

This review aims to provide a detailed and systematic examination of the molecular design strategies employed in the development of STING agonists. It particularly focuses on key structure–activity relationship (SAR) findings relevant to STING‐targeted therapeutics in cancer treatment. By integrating chemical, biological, and pharmacological insights, this work seeks to offer a comprehensive perspective to support the future design and optimization of more effective STING agonists. This manuscript is a review article and does not include any new original experimental data.

## 2 Mechanisms and Functions of the cGAS‐STING Signaling Pathway in Immune Activation

The cGAS‐STING pathway is a crucial component of the innate immune system that senses cytosolic dsDNA derived from pathogens, damaged mitochondria, or the host's cells. cGAS recognizes dsDNA in a length‐dependent but sequence‐independent manner, forming a 2:2 complex with DNA that induces conformational changes in cGAS. These changes enable cGAS to enzymatically produce the second messenger 2′3′‐cyclic GMP‐AMP (2′3′‐cGAMP) from ATP and GTP (Zheng et al. 2020).

2′3′‐cGAMP binds to STING, an ER‐associated protein, causing STING to oligomerize and translocate to the Golgi apparatus (Hopfner and Hornung 2020). There, STING is palmitoylated, a modification necessary for its activation. Activated STING recruits and activates TBK1, which first phosphorylates STING to create a docking site for IRF3, and subsequently phosphorylates the recruited IRF3. Phosphorylated IRF3 dimerizes and translocates to the nucleus, where it drives the expression of IFN‐Is and interferon‐stimulated genes. Concurrently, STING activates the NF‐κB pathway, promoting the production of inflammatory cytokines such as tumor necrosis factor alpha (TNF‐α) and IL‐6 (Zhong et al. 2024). This signaling cascade initiates strong innate immune responses and primes adaptive immunity, contributing to antiviral defense, inflammation, and antitumor effects. Signal termination is achieved by trafficking STING to endolysosomes for degradation. Furthermore, cGAMP can spread between cells via gap junctions or be delivered in extracellular vesicles, amplifying immune activation beyond the initially affected cells. Thus, the cGAS‐STING axis functions as a key sensor and mediator linking detection of cytosolic DNA to immune defense mechanisms.

### 2.1 STING Pathway in Antitumor Immunity

The STING pathway is a key mediator of antitumor immunity, linking innate and adaptive responses. Upon sensing cytosolic DNA, STING activation induces type I interferons and proinflammatory cytokines, enhancing dendritic cell function, CD8^+^ T cell priming, NK cell cytotoxicity, and macrophage activation. These coordinated responses reshape the tumor microenvironment, recruit immune cells, and promote robust antitumor activity, making STING a promising target for cancer immunotherapy (Li et al. 2025).

### 2.2 Mechanisms of STING Activation by Tumor‐Derived DNA

In healthy cells, DNA is confined to the nucleus and mitochondria to avoid triggering immune responses. However, tumor cells experience genome instability, mutations, and metabolic stress, which cause DNA to leak into the cytoplasm. This leaked DNA can take several forms, including micronuclei and chromatin fragments, often resulting from errors during cell division. This cytoplasmic DNA activates the cGAS‐STING pathway (Li et al. 2024). Additional DNA sources that activate STING include mitochondrial DNA released due to oxidative stress or damage from treatments like chemotherapy and radiation, apoptotic cell DNA, and DNA contained within exosomes or transposable elements (McAndrews et al. 2021).

### 2.3 Role of Type I Interferons in Antitumor Immunity

When STING is activated, it triggers the production of type I IFNs, which play important roles in controlling tumor growth. Inside tumor cells, this activation can cause cellular senescence, a permanent stop in cell division, and it can promote programmed cell death by regulating both proapoptotic and antiapoptotic factors (Liang et al. 2021).

STING activation in nearby non‐tumor cells also supports tumor suppression by enhancing tissue repair and stimulating immune responses. Specialized immune cells known as DCs engulf dying tumor cells and present tumor antigens to CD8^+^ T cells. Type I IFNs assist in maturing DCs, improving their ability to present antigens, and promoting the recruitment of cytotoxic T cells to the tumor site. Additionally, type I IFNs reduce the activity of regulatory T cells that normally suppress immune responses, thereby strengthening the immune system's attack on cancer (Zaidi et al. 2021).

### 2.4 Ligand‐Independent Non‐Canonical STING Signaling Pathways

Beyond its established role in the canonical cGAS‐cGAMP‐STING‐TBK1‐IRF3 pathway, cGAMP also mediates noncanonical STING‐dependent signaling pathways that are independent of type I interferon induction. cGAMP‐induced STING activation has been shown to promote autophagy through Microtubule‐associated protein 1 light chain 3B (LC3B) (also known as MAP1LC3B) lipidation, with STING functioning as a proton channel to facilitate autophagosome biogenesis (Zhang and Zhang 2025). In parallel, STING activation in human monocytes can prime lysosomal cell death and NOD‐like receptor family pyrin domain containing 3 (NLRP3) inflammasome activation, highlighting a direct link between cGAMP‐STING signaling and inflammatory cell death pathways. Additionally, cGAMP‐activated STING initiates a noncanonical STING‐Protein kinase R‐like endoplasmic reticulum kinase (PERK)‐eukaryotic Initiation Factor 2 alpha (eIF2α) signaling axis, distinct from the classical TBK1‐IRF3 cascade. In this pathway, ER‐resident STING directly activates PERK, leading to eIF2α phosphorylation and translational reprogramming that favors inflammatory and survival responses. This signaling occurs prior to STING translocation to the Endoplasmic‐reticulum‐Golgi intermediate compartment (ERGIC)/Golgi and may contribute to cellular senescence and organ fibrosis. Collectively, these findings demonstrate that cGAMP functions as a versatile second messenger, orchestrating multiple STING‐dependent signaling outputs beyond interferon production, thereby expanding its physiological and therapeutic relevance (Chen, Yue, et al. 2025). While these noncanonical pathways were initially identified through cGAMP‐mediated activation, it is important to note that they are fundamentally linked to the conformational rearrangement and translocation of the STING protein. Therefore, synthetic noncyclic dinucleotide (non‐CDN) agonists that induce similar structural shifts and ER‐to‐Golgi trafficking are also expected to trigger these noncanonical responses, potentially contributing to their antitumor efficacy independently of the IRF3‐IFN axis.

## 3 Mechanisms of STING Activation and Ligand Binding

STING is an integral membrane protein located on the ER that is essential for activating innate immune responses and inhibiting tumor growth. It forms a homodimer shaped like a “V,” with each unit made up of 379 amino acids (Li et al. 2025). The protein consists of an N‐terminal region containing four transmembrane helices and a connecting helix, along with a C‐terminal region that houses the ligand‐binding domain and a flexible tail. STING can exist in two main shapes: an “open” form where the binding pocket is exposed without a covering structure, and a “closed” form in which a β‐sheet lid folds over the pocket to stabilize ligand binding (Lu et al. 2022). When natural molecules like 2′,3′‐cGAMP bind, the ligand‐binding domain undergoes a large rotation relative to the transmembrane portion, breaking internal interactions and encouraging the formation of tetramers and larger complexes (Shang et al. 2019). This change also frees the C‐terminal tail (CTT), triggering downstream signaling pathways such as IRF3 phosphorylation, NF‐κB activation, and the release of type I interferons (Liu et al. 2015). Molecular dynamics simulations have provided further insights into the binding and conformational dynamics of STING upon ligand interaction. Studies involving cyclic adenosine‐inosine monophosphate (cAIMP) analogs showed that these molecules adopt a U‐shaped configuration in the STING binding pocket. Key residues such as Arg238, Thr263, Thr267, Ser162, Tyr167, and Tyr240 play crucial roles in stabilizing the ligand‐protein complex (Ouyang et al. 2012). Arg238 forms strong hydrogen bonds with high occupancy, acting as a major anchor point. Thr263 and Thr267 are also consistently involved in hydrogen bonding, contributing significantly to binding stability in both open and closed STING states. Additionally, Tyr167 engages in π‐π stacking interactions with the ligand bases, while hydrophobic residues like Val239 and Pro264 provide further stabilizing interactions.

**FIGURE 2:**
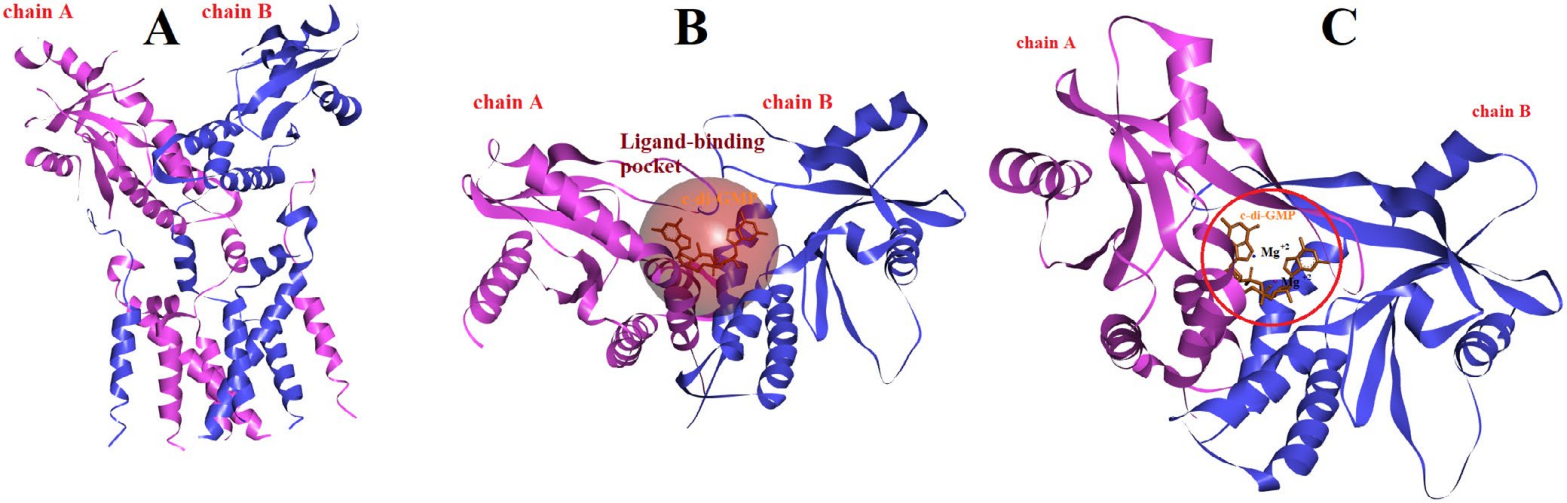
Structural representations of human STING (hSTING) in different conformational states and ligand‐bound forms. The A and B chains are colored pink and blue, respectively. (A) Depicts the full‐length hSTING structure (PDB ID: 6NT5). (B) Structure of the hSTING C‐terminal domain (CTD) dimer bound to the cyclic dinucleotide c‐di‐GMP (PDB ID: 4F5Y), with the ligand positioned at the dimer interface, stabilizing STING and illustrating the ligand‐binding pocket. (C) Crystal structure of the hSTING CTD dimer in the ligand‐bound closed conformation, complexed with c‐di‐GMP (PDB ID: 4F5D).

Interestingly, some non‐nucleotide agonists, such as amidobenzimidazole derivatives, can activate human STING (hSTING) without inducing complete lid closure, suggesting an alternative mechanism of activation in a partially open state. Comparative simulations also reveal that ligands bound to the closed conformation generally exhibit a tighter and more folded U‐shape, often forming more hydrogen bonds and demonstrating stronger binding affinities than in the open state. However, certain modified ligands like cAIMP3 and cAIMP5 have shown stable and deep binding even in the open conformation, indicating their potential for strong activation despite the absence of full lid formation (Figure 2). Together, these structural and dynamic insights highlight the complexity of STING's activation mechanism and underscore the importance of specific amino acid interactions in ligand binding and signal initiation (Wang et al. 2024). Molecular docking poses were visualized with BIOVIA Discovery Studio Visualizer.

## 4 CDN and Non‐CDN STING Agonists

STING agonists represent a new class of immunotherapies that activate the immune system to fight tumors. These compounds stimulate the STING pathway, leading to interferon production and enhanced antitumor responses. They are grouped into CDN and non‐CDN agonists, with each group offering unique structural characteristics and therapeutic advantages.

### 4.1 CDN STING Agonists

**FIGURE 3:**
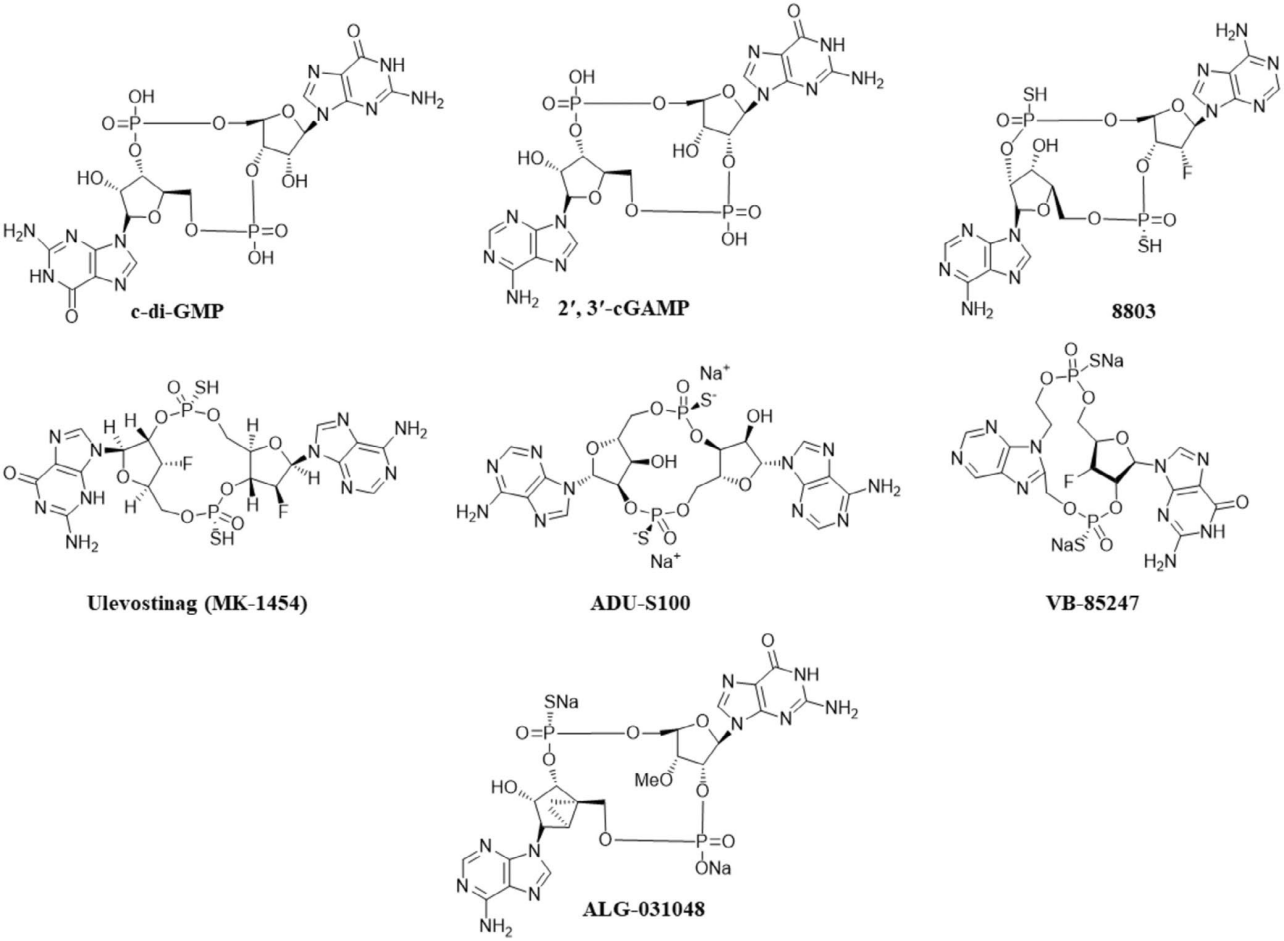
CDNs and CDN derivatives.

CDN analogs are synthetic compounds designed to replicate the function of natural CDNs, such as **2′,3′‐cGAMP**, which plays a key role in activating the STING signaling pathway. By directly binding to the STING receptor, these molecules initiate downstream immune responses crucial for antitumor immunity. Natural CDNs like **2′,3′‐cGAMP** and c‐di‐GMP can activate STING, but they are limited by poor metabolic stability and restricted cellular uptake (Figure 3). To overcome these barriers, **ADU‐S100** (**MIW815**) and **Ulevostinag** (**MK‐1454**) were developed as synthetic CDN analogs with improved pharmacological properties. **ADU‐S100**, developed through a collaboration between Aduro Biotech and Novartis, entered clinical development following a 2015 partnership to advance Aduro's preclinical STING assets. About a year later, **ADU‐S100** entered Phase 1 clinical trials for various solid tumors. Structurally, **ADU‐S100** differs from natural cGAMP through the replacement of both phosphodiester linkages with phosphorothioate bonds, a modification commonly found in antisense oligonucleotide therapeutics. This design confers enhanced metabolic stability and improved cellular uptake, making **ADU‐S100** more suitable for therapeutic use. Similarly, **Ulevostinag**, developed by Merck, incorporates analogous modifications and has demonstrated promising antitumor activity, particularly when combined with immune checkpoint inhibitors (Maddess et al. 2022). **8803** (**IMGS‐203**) is a synthetic CDN STING agonist that outperforms natural CDNs and the clinical benchmark **ADU‐S100** in activating STING and inducing antitumor immunity in vitro and in vivo. The exact mechanism of tumor regression remains under investigation (Jiang and Fei 2025). **ALG‐031048** is a novel 2′3′‐CDN STING agonist with a chemical structure consisting of an adenosine monomer with a north‐methanocarba sugar modification and a 3′‐*O* methyl‐modified guanosine monomer, connected by single phosphorothioate and phosphodiester bonds (Amouzegar et al. 2021). **VB‐85247**, a CDN analogue developed by Venenum Biodesign in 2021, activates the STING pathway to stimulate innate immune responses and suppress tumor growth (Zhao et al. 2025).

### 4.2 Non‐CDN STING Agonists

**FIGURE 4:**
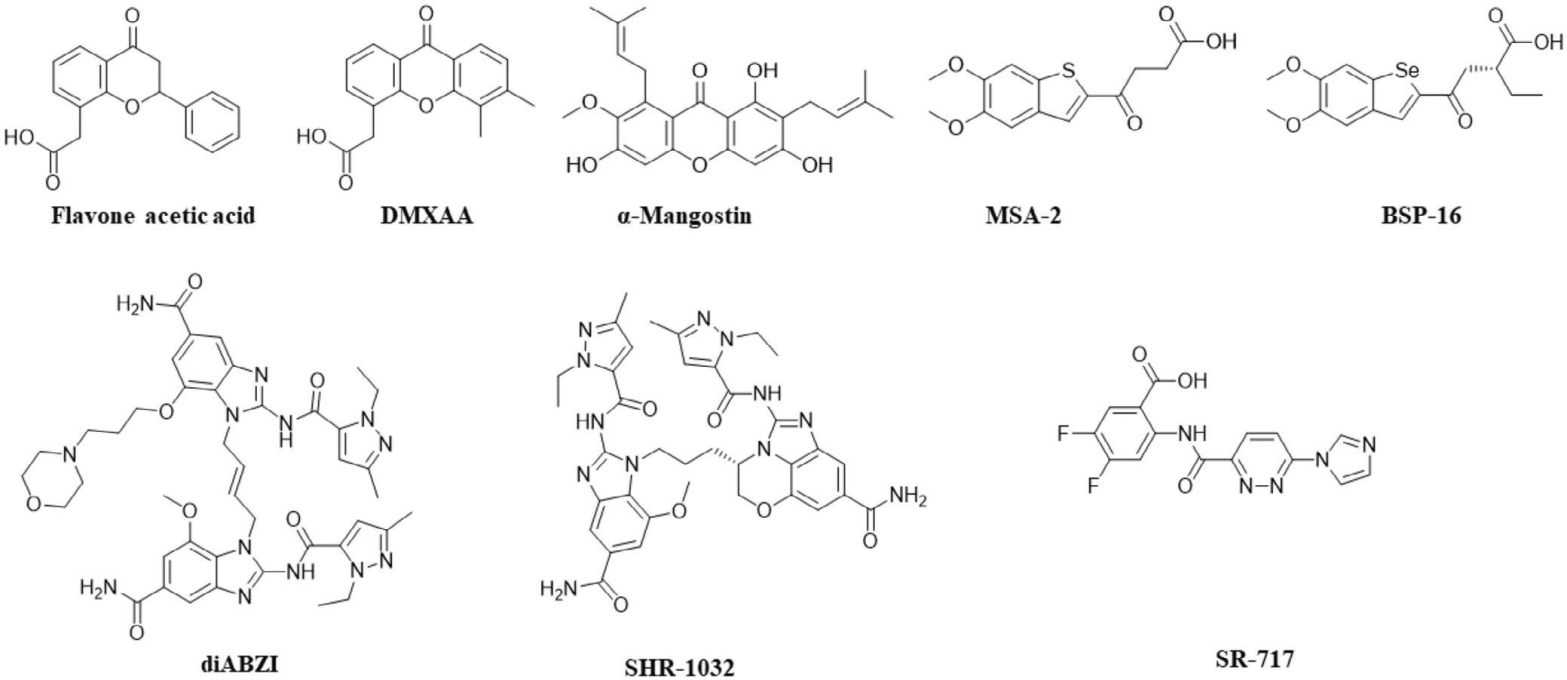
Non‐CDN STING agonists.

Non‐CDN STING agonists have emerged as promising alternatives to overcome the limitations of CDNs (Qu and Dai 2025). One of the earliest examples is **flavone acetic acid** (**FAA**), initially used as a vascular‐disrupting agent that induced hemorrhagic necrosis in murine tumors. However, its clinical application was limited due to a narrow therapeutic window and poor pharmacokinetic properties. 5,6‐dimethylxanthenone‐4‐acetic acid (**DMXAA**) is a chemotherapeutic agent that exhibits strong antitumor effects in mouse tumor models by disrupting tumor vasculature, inducing nitric oxide production from tumor‐associated macrophages, and stimulating the production of tumoricidal cytokines, such as TNF‐α and type I interferons, in a STING‐dependent manner. However, its antitumor efficacy was limited in humans, as it failed in phase III clinical trials for nonsmall cell lung cancer (NSCLC). This failure was later attributed to **DMXAA**'s inability to activate the hSTING pathway, as it only activates the mouse STING pathway (Temizoz et al. 2024). **α‐Mangostin**, a natural product isolated from mangosteen, shares the anthrone scaffold (Yu et al. 2024) with **DMXAA** and demonstrated higher activity toward hSTING than murine STING (mSTING), laying the groundwork for the rational design of new hSTING‐selective agonists. **DiABZI**, short for diamidobenzimidazole (Nguyen et al. 2024), is a potent non‐CDN STING agonist designed to overcome the limitations of natural CDNs, such as poor cellular permeability and the requirement for intratumoral administration. The small non‐CDN molecule **diABZI** binds to the cytosolic nucleotide‐binding domain of STING and, unlike **2′,3′‐cGAMP**, promotes dimerization while maintaining STING in an open conformation. This unique binding mechanism allows for potent activation of the hSTING pathway without structurally mimicking cyclic dinucleotides (CDNs) (Qu and Dai 2025). Its dimeric amidobenzimidazole scaffold enables strong and selective STING activation, making it a promising candidate for systemic use in cancer immunotherapy (Ramanjulu et al. 2018). Among other next‐generation synthetic agonists, **MSA‐2** is an orally available, noncovalent dimeric STING agonist that preferentially activates STING in tumors, inducing robust antitumor immunity and showing potential for systemic cancer therapy (Pan et al. 2020). **SHR1032** has shown high potency across hSTING isoforms, inducing strong IFNβ responses and promoting apoptosis in acute myeloid leukemia cells (Heery et al. 2019). **SR‐717** has demonstrated strong antitumor activity in preclinical models by enhancing the function of CD8^+^ T cells, NK cells, and DCs, while also promoting antigen presentation and PD‐L1 expression in a STING‐dependent manner (Chin et al. 2020). **BSP‐16** is a non‐CDN STING agonist structurally derived from **MSA‐2**. By replacing the sulfur atom in **MSA‐2**'s benzothiophene ring with selenium, researchers created a benzoselenophene (BSP) scaffold and introduced further modifications at the C2 and C3 positions (Figure 4). Among the resulting compounds, **BSP‐16** showed superior STING activation, better solubility, membrane permeability, and oral bioavailability (107%) (Feng et al. 2022). These non‐CDN agonists represent diverse structural and mechanistic approaches aimed at achieving selective, effective, and safe activation of hSTING for cancer immunotherapy (Yu et al. 2024).

### 4.3 Modulating STING Oligomerization: A Novel Design Strategy

The fundamental trigger for STING activation extends beyond the mere binding of a ligand to the dimeric pocket; it relies heavily on the subsequent formation of high‐order protein clusters, known as oligomerization. STING is a 379‐amino acid transmembrane protein localized in the ER, consisting of four transmembrane domains (TMDs) and a cytoplasmic ligand‐binding domain (LBD). In its resting state, STING remains in an autoinhibited conformation, maintained by intramolecular interactions involving its CTT.

As comprehensively reviewed by Chen, Yue, et al. (2025) and Chen, Zhuo, and Li (2025), the binding of cGAMP or synthetic small‐molecule agonists induces a dramatic 180° rotation of the LBD relative to the TMD. This conformational shift rearranges the connector‐LBDα_1_ elements, transitioning them from a right‐handed crossover to a parallel configuration. This structural reorganization exposes lateral interfaces that facilitate the side‐by‐side packing of STING dimers into tetramers and larger oligomeric assemblies. This clustering is a mandatory prerequisite for the translocation of STING from the ER to the Golgi apparatus, where it undergoes essential posttranslational modifications, such as palmitoylation at Cys88/91. These palmitoylated clusters serve as a high‐affinity platform for the recruitment and trans‐autophosphorylation of TBK1 and the subsequent activation of IRF3.

From a medicinal chemistry perspective, targeting STING oligomerization offers a sophisticated “pocket‐independent” design strategy. Traditional agonists often face translational challenges due to species‐specific differences between murine and hSTING, as well as genetic polymorphisms (e.g., H232 vs. R232 variants) within the human population. In contrast, emerging chemical strategies including the development of multivalent polymers, polypeptides, or outer‐pocket regulators can directly induce or stabilize STING oligomerization without relying solely on the central binding cavity. By lowering the threshold for higher order assembly, these oligomerization‐driven agonists can elicit robust immune responses even in contexts where traditional CDNs show limited efficacy. This shift toward rational control of protein assembly represents a pivotal advancement in overcoming the current limitations of STING‐targeted immunotherapies (Chen, Zhuo, and Li 2025).

## 5 Preclinical Insights into STING Agonists: Limitations and Therapeutic Potential

The therapeutic potential of STING agonists in cancer remains under investigation, and their clinical prospects are currently uncertain. Intratumoral (IT) administration offers a targeted approach with a reduced risk of systemic cytokine storms; however, it is limited by tumor heterogeneity, the rapid diffusion and short half‐life of CDNs, and the need for repeated injections, as observed in studies using **ADU‐S100** in melanoma models. Systemic administration, on the other hand, can potentially reach inaccessible tumors but carries significant risks, including offtarget STING activation, severe cytokine release syndrome, autoimmunity, and pro‐tumor inflammatory responses. For instance, systemic **MSA‐2** administration in preclinical models raised concerns about activation in non‐tumor tissues, highlighting the need for careful safety evaluation. Additionally, hSTING genetic variants exhibit differential responsiveness, such that compounds like **diABZI** show variable potency depending on the STING isoform, emphasizing the importance of considering both variant type and geographic distribution in clinical trial design.

Despite these limitations, STING agonists continue to demonstrate promising therapeutic effects in preclinical models. Compounds such as **8803**, **VB‐85247**, and **ALG‐031048** induce type I interferons, enhance CD8^+^ T cell responses, and establish durable immune memory. Notably, these effects lead to significant tumor regression in glioblastoma, colon carcinoma, melanoma, and AML models, even for compounds characterized by modest binding affinity like **ALG‐031048**. Systemically administered diABZI shows strong innate immune activation and synergizes with anti‐PD‐1 therapy. At the same time, oral **MSA‐2** selectively activates the STING pathway in the acidic tumor microenvironment, promoting IFN‐β secretion and tumor cell apoptosis with minimal systemic toxicity. **SR‐717**, delivered intraperitoneally, increases CD8^+^ T cell and NK cell infiltration into tumor‐draining lymph nodes and enhances antitumor responses in melanoma models. Additional benefits observed across studies include modulation of tumor vasculature, upregulation of endothelial‐leukocyte adhesion molecules, induction of anti‐angiogenic factors such as CXCL10, and increased infiltration of effector immune cells, collectively contributing to improved antitumor efficacy. Next‐generation STING agonists, compared with earlier agents such as **ADU‐S100** and **Ulevostinag**, are designed to bind across all major hSTING isoforms, exhibit resistance to Ectonucleotide pyrophosphatase/phosphodiesterase 1 (ENPP1)‐mediated degradation, and display improved chemical stability and optimized pharmacokinetic properties. As a result, they induce stronger type I IFN production while reducing toxicity, thereby offering the potential for effective systemic administration. Taken together, these findings support the continued development of STING agonists as promising immunotherapeutic agents, provided that delivery strategies, dosing regimens, and safety profiles are carefully optimized regimens (Table 1) (Shi et al. 2025).

**TABLE 1:** Preclinical studies of STING agonists.

Compounds	Drug types	Study type/Route of administration	Animal models used	Cancer type	Benefits	Limitations	References
8803	CDN STING agonist (Najem et al. 2024a, 2024b)	Preclinical studies ic	Murine orthotopic glioblastoma models (QPP4, QPP8)	Immune checkpoint‐resistant glioblastoma	Long‐term survival in 56%–100% of treated mice (*p* = 0.0003 to < 0.0001) Maintained efficacy in humanized mice (even with tumor STING silencing) Increased IFN release and tumor immune effector trafficking	Epigenetic silencing of STING in human glioblastoma may limit translatability Optimal imaging time window (48–96 h posttreatment) must be strictly followed Imaging and voxel‐wise analysis require advanced techniques and resources Human toxicity/safety unknown	Najem et al. (2024a, 2024b)
VB‐85247	Macrocyclic STING agonists (Hao et al. 2023)	Preclinical Intravesical	Orthotopic NMIBC model	NMIBC	100% complete response at 40 μg dose in mice Tumor regression observed after just 1 dose Immunologic memory (rechallenge: tumor rejection) Induces IFNβ, CXCL10, CCL5, TNFα, IL6 Activates systemic immune response	2‐h exposure required for activity—shorter durations ineffective Human clinical safety yet unproven Repeated stimulation may lead to immune exhaustion/toxicity (needs monitoring) No human efficacy data yet (clinical trials needed)	Prabagar et al. (2025)
VB‐85247+ Pembrolizumab	Macrocyclic STING agonists anti‐PD‐1 immuno‐oncology agent	Preclinical combination study VB‐85247 Intravesical, Pembrolizumab ip	NMIBC (MB49‐luc mouse)	Anti‐PD‐1 resistant NMIBC	100% complete response in combination group Better than monotherapy with either agent alone More effective in anti‐PD‐1 resistant tumors Faster tumor regression	Dosing and scheduling must be optimized in clinical trials Human safety profile of the combination is unknown Potential for autoimmune‐related toxicities	Prabagar et al. (2025)
VB‐85247 vs. BCG (Bacillus Calmette‐Guérin) (comparative)	CDN STING agonist Zhao et al. (2025)	Preclinical VB‐85247 intravesical BCG intravesical	NMIBC MB49‐luc mouse	BCG‐unresponsive NMIBC	VB‐85247: 9/10 mice survived with tumor regression BCG: 1/10 mice survived Faster and more robust antitumor activity with VB‐85247	BCG is ineffective in this mouse model, limiting direct clinical translation Combination with BCG not recommended based on current data	Prabagar et al. (2025)
ALG‐031048	CDN STING agonist Amouzegar et al. (2021)	Preclinical IT	CT26 (colon carcinoma), B16F10 (melanoma) mouse model	Colon carcinoma, melanoma	STING binding (The equilibrium dissociation constant (K_D_) 3.12 μM, thermal shift 12.8°C) Stronger IFNβ/IRF activation than **ADU‐S100** High stability (no degradation in SVPD) Superior in vivo tumor regression (90% at 100 μg IT) Protective immune memory upon tumor re‐challenge	Data only from preclinical models No human safety or pharmacokinetic (PK) data yet Immune response heterogeneity between tumor models not deeply explored	Jekle et al. (2020)
8803	CDN STING agonist	Preclinical IT	CT26 (colon), B16F10 (melanoma), humanized GBM	Solid tumors, Glioblastoma	Potent STING activation 100% tumor clearance in CT26 Improved survival in B16F10 Immune‐mediated killing with PBMCs	Human safety/toxicity unknown IT delivery may limit systemic use	Salameh et al. (2024)
SHR1032	Non‐CDN STING agonist Song et al. (2023)	Preclinical IT	MC38 syngeneic (C57BL/6 mice), THP1, MV4‐11, MOLM‐16	Solid tumors, AML	High potency in hSTING isoforms Strong IFNβ induction Tumor growth inhibition (78%) Induces direct AML cell apoptosis	Lower activity in mSTING cells Still early‐stage	Song et al. (2022)
ALG‐031048	CDN STING agonist	Preclinical intratumoral subcutaneous	CT26 (colon), Hepa1‐6 (liver), colon adenocarcinoma cells expressing human programmed death‐ligand 1 (MC38‐hPD‐L1)	Colon carcinoma Hepatocellular carcinoma	Demonstrated antitumor activity despite being a weak binder Up to 90%–100% complete tumor regression (IT) Long‐lasting, antigen‐specific immune memory Active after systemic (sc) administration Synergistic with immune checkpoint inhibitors (ICIs), such as anti‐cytotoxic T‐lymphocyte‐associated protein 4 (anti‐CTLA‐4) and anti‐programmed death‐ligand 1 (anti‐PD‐L1) Improved stability vs. first‐generation CDNs	Optimal therapeutic outcomes are primarily achieved with IT dosing Systemic efficacy requires higher sc doses Data limited to subcutaneous tumor models Orthotopic models not yet tested	Jekle et al. (2023)
LCB39	Non‐CDN STING agonist Oh et al. (2025)	Preclinical systemically	THP1‐Dual Cells (in vitro) Syngeneic mouse models: CT26, 4 T1 (Balb/c), MC38, B16F10 (C57BL/6) Preliminary monkey NHP study	Various solid tumors (colon, breast, melanoma)	Tumor‐targeted with retention Robust antitumor activity dose‐dependently High tolerability and low cytotoxicity to normal cells Promotes immune priming and antitumor memory CD8 T cells Favorable safety profile in preliminary NHP studies	Lower direct binding potency and in vitro reporter activity compared to some analogs Fast clearance may require optimized dosing Preclinical stage; clinical efficacy and safety yet to be confirmed	Oh et al. (2024)
ADU‐S100	CDN STING agonists	Preclinical IT	B16 melanoma, BPR20 mouse model	Melanoma	Slows tumor growth, Induces immune activation, Increases inflammatory signaling	B16 is derived from a male mouse, whereas BPR20 is derived from a female mouse Induces regulatory ISGs‐including PD‐L1, ISG15, ARG2, NOS2, and COX2 which collectively create a feedback loop that limits therapeutic efficacy	Filderman et al. (2024)
Ulevostinag	CDN‐ STING agonists Huang et al. (2022)	Preclinical IT	MC38 (colorectal) and B16‐F10 (melanoma) mouse tumor model	Colorectal adenocarcinoma, melanoma	Potent in cell‐based assays, similar binding characteristics as 2′3′‐cGAMP, reduced tumor volume in MC38 and B16‐F10 mouse tumor model	Ulevostinag is a highly polar cyclic dinucleotide, which limits: Membrane permeability Systemic exposure Tissue penetration	Gehrcken et al. (2025)

Abbreviations: ARG2, arginase 2; COX2, cyclooxygenase 2; ic, intracranial; ip, intraperitoneal; ISG15, interferon‐stimulated gene 15; ISGs, interferon‐stimulated genes; IT, intratumoral; Intravesical; mSTING, mouse STING; NHP, non‐human primate; NMIBC, non‐muscle‐invasive bladder cancer; NOS2, nitric oxide synthase 2; sc, subcutaneous.

### 5.1 Overcoming STING Agonist Challenges

Despite their antitumor potential, STING agonists used as monotherapy and via intratumoral administration face key limitations, including poor tumor penetration, rapid enzymatic degradation, short half‐life, and the need for repeated dosing, all of which can reduce clinical effectiveness. To overcome these challenges, advanced delivery strategies such as nanocarriers and antibody‐drug conjugates (ADCs) have been developed to enhance efficacy, improve bioavailability, and minimize systemic toxicity (Gehrcken et al. 2025).

Nanocarriers, including liposomes, polymersomes, peptide nanoparticles, and lipid nanoparticles, enable targeted systemic delivery and prolong agonist half‐life. For example, a biopolymer containing a STING agonist and natural killer group 2D (NKG2D)‐based chimeric antigen receptor (CAR) T cells applied to a pancreatic tumor in mice efficiently delivered CAR‐T cells and transformed the tumor bed into a “self‐vaccine site.” (Smith et al. 2017). Encapsulated cGAMP combined with cytosine‐phosphoguanine oligodeoxynucleotides (CpG ODN) and the model antigen ovalbumin (OVA) enhanced T helper type 1 (Th1) immune responses in vitro, reduced M2‐polarized macrophages, and decreased melanoma tumor volume by up to 70% in vivo (Gehrcken et al. 2025). In glioblastoma models, bridging‐lipid nanoparticles targeting CD47/PD‐1 on tumor‐associated myeloid cells, combined with **diABZI**, reprogrammed immunosuppressive cells into an anti‐tumoral phenotype (Zhang et al. 2023). Nakamura et al. (2021) demonstrated that STING‐loaded lipid nanoparticles enhanced NK cell activity in a B16‐F10 melanoma model, with tumor control mediated primarily by NK cells. ADCs provide an additional strategy to achieve tumor‐specific STING activation while reducing systemic side effects. EGFR targeted cGAMP analogue **IMSA172** (Huang et al. 2024) ADCs showed exceptionally potent and antigen dependent STING activation, more than 10,000 fold stronger than free **IMSA172**, demonstrating that targeted delivery can effectively overcome the limitations of systemic STING agonists (Wu et al. 2022). Despite current clinical uncertainties, STING agonists exhibit significant antitumor potential, and advanced delivery strategies such as nanocarriers and ADCs offer promising avenues to enhance efficacy and safety, warranting further preclinical and clinical evaluation.

### 5.2 STING Agonist Chemotherapy Combinations

Combination strategies are increasingly explored to overcome the limited efficacy of STING agonists when used alone. In several preclinical studies, camptothecin‐STING agonist hydrogels produced markedly stronger tumor suppression compared with either monotherapy, demonstrating that targeted DNA damage can act in synergy with STING activation to amplify antitumor immunity. Likewise, systemic nanosystems co‐delivering **SN38** and **DMXAA** achieved superior tumor control relative to free **DMXAA** or chemotherapy alone. This synergy is not merely a dual activation of the same signaling axis; rather, it arises because chemotherapy‐induced genotoxic stress triggers immunogenic cell death (ICD), which primes the tumor microenvironment by increasing the visibility of tumor antigens and recruiting immune cells. While the endogenous cGAS‐mediated response to chemotherapy is often minor or defective in many tumors, the concurrent administration of a direct STING agonist provides a potent boost. This allows the therapy to effectively bypass the often‐impaired upstream cGAS signaling and overcome the local immunosuppressive barriers, ensuring a robust and sustained T‐cell response. Together, these findings support combination‐based approaches as a promising means to enhance the therapeutic impact of STING agonists (Du et al. 2025; Shang et al. 2026; Singhai et al. 2026).

## 6 Clinical Implications, Limitations, and Advances in STING Agonist Therapies

The cGAS‐STING pathway is increasingly recognized as a critical tumor suppressor, and its reduced expression correlates with poor prognosis in various malignancies. Consequently, this pathway remains a pivotal therapeutic target, prompting the development of multiple classes of STING agonists, includingCDNs, **DMXAA** analogs, and small‐molecule activators. However, despite promising preclinical results, the translation of these agents into clinical practice has faced significant challenges.

### 6.1 Limitations of First‐Generation Agonists

Initial efforts to target STING encountered major hurdles, primarily due to species‐specific activity. A prominent example is the flavonoid **DMXAA**, which failed in clinical trials because it effectively activates mSTING but fails to bind hSTING. Subsequent CDN agonists, although potent in vitro, suffer from intrinsic pharmacological limitations. These include rapid enzymatic degradation by phosphodiesterases, poor membrane permeability due to anionic phosphate groups, and the risk of systemic inflammatory toxicity (Lu et al. 2025). Clinically, monotherapy responses have been modest, with many patients achieving only stable disease rather than tumor regression, highlighting the need for optimized therapeutic strategies.

### 6.2 Overcoming Pharmacological Barriers: The Emergence of Non‐CDN Agonists

To overcome the bioavailability and stability issues of natural CDNs, non‐CDN STING agonists have emerged as versatile clinical candidates. Unlike natural CDNs, non‐CDN small molecules offer greater chemical flexibility, allowing for the optimization of metabolic stability and oral bioavailability. While this structural versatility enables the fine‐tuning of potent interactions with hSTING, it is important to note that high species‐selectivity often presents a significant challenge in drug development. Many non‐CDN agonists exhibit limited cross‐species activity, which complicates preclinical testing by restricting their evaluation for efficacy and safety within standard murine models. Despite these translational hurdles, **ABZI** derivatives represent a significant advancement in systemic delivery, generating robust type I interferon responses with improved pharmacokinetics compared to traditional CDNs. Furthermore, **MSA‐2** has introduced a breakthrough in tumor‐targeted activation as an orally bioavailable agonist; it achieves effective intratumoral stimulation through pH‐dependent dimerization within the acidic tumor microenvironment, thereby minimizing systemic toxicity. The structural versatility of this class is further demonstrated by novel scaffolds such as **α‐Mangostin** derivatives, which can be tuned to target specific hSTING variants, potentially allowing for personalized approaches to immunotherapy (Huang et al. 2022; Shen et al. 2026).

### 6.3 Combination‐Based Therapeutic Strategies

Given that STING agonists as monotherapy often demonstrate limited clinical efficacy, combination strategies have become essential to maximize therapeutic outcomes. STING activation is increasingly paired with established modalities such as chemotherapy, radiotherapy, and particularly immune checkpoint inhibitors (Wang et al. 2025). The rationale behind combining STING agonists with anti‐PD‐1/PD‐L1 antibodies lies in their ability to overcome adaptive immune resistance by converting “cold” tumors into “hot” tumors through enhanced T‐cell infiltration and type I IFN production. Recent clinical data summarized in Table 2 underscore the shift toward these synergistic regimens. Notably, the non‐CDN STING agonist **MK‐2118** represents a novel mechanistic approach in this field. As a structural relative of **MSA‐2, MK‐2118** exhibits a unique pH‐dependent activity, functioning as a molecular “glue” that promotes STING dimerization specifically within the acidic tumor microenvironment. This targeted mechanism of action potentially mitigates systemic toxicity while enhancing localized immune activation. Currently, **MK‐2118** is being evaluated in Phase I trials both as a monotherapy and in combination with **pembrolizumab** (Luke, Sweis, et al. 2025). While **MK‐2118** alone showed limited objective responses, its combination with **pembrolizumab** achieved an overall response rate (ORR) of 3.8%–6.0% in patients with advanced solid tumors, including melanoma and cutaneous T‐cell lymphoma, supported by increased levels of IFN‐γ and interferon‐gamma‐induced protein 10 (IP‐10) (Wang et al. 2020). Furthermore, STING activation can synergize with cytotoxic agents. For instance, camptothecin‐STING agonist hydrogels and systemic nanosystems co‐delivering **SN38** and **DMXAA** have demonstrated superior tumor control by simultaneously inducing DNA damage and STING‐mediated cytokine release (Liang et al. 2020; Zhao et al. 2021). Collectively, these advancements confirm that the future of STING‐targeted therapy lies in integrating potent agonists, particularly clinically validated non‐CDN molecules like **MK‐2118**, which stands as the first of its class to demonstrate clinical efficacy, into synergistic combination regimens (Le Naour et al. 2020).

**TABLE 2:** Clinical studies and therapeutic agents targeting the STING pathway in cancer.

Drug/Combination	Drug class	Study/Trial	Patient population	Cancer type	Therapy benefits	Adverse events	References
TAK‐676	CDN STING agonist	Phase I	36 patients	Colorectal and head and neck cancer	Linear pharmacokinetics across the dose range tested	Fatigue (33%), Nausea (29%), Pyrexia (22%), Increased risk of fever/cytokine release syndrome with higher exposure	Olszanski et al. (2023)
TAK‐676 Pembrolizumab	CDN STING agonist, PD‐1 inhibitor	Phase I	43 patients	Colorectal and head and neck cancer	Increased the number of Ki67^+^CD8^+^ T cells, Increased production of IFN‐γ and IP‐10.	Enhances adaptive immune response Fatigue, Nausea	Olszanski et al. (2023)
IMSA101	CDN STING agonist	Phase I	Adults with advanced solid tumors, ≥ 2 evaluable lesions (≥ 1 injectable)	Advanced solid tumors (including NSCLC, melanoma, RCC)	Well‐tolerated at 1200 μg No plasma accumulation Short half‐life (1.5–2 h)	Injection site pain (36.4%), Fatigue (18.2%)	Jacoby et al. (2025)
ADU‐S100	CDN STING agonist	Phase I	Adults with advanced/metastatic solid tumors or lymphomas, ECOG 0–1, refractory/intolerant to standard therapy	Various solid tumors and lymphomas (including melanoma, breast, colorectal, Merkel cell carcinoma, sarcoma)	Well tolerated Partial response in 1 patient (Merkel cell carcinoma) Stable/decreased size in 94% of injected lesions Evidence of systemic immune activation (cytokines, T‐cell expansion)	Pyrexia (17%), Chills (15%), Injection‐site pain (15%) Grade 3/4 AEs in 40% (anemia, hyponatremia most frequent)only one dose‐limiting toxicity (injection site ulcer)	Meric‐Bernstam, Sweis, Hodi, et al. (2023)
ADU‐S100 + Spartalizumab (PDR001)	CDN STING agonist PD‐1 inhibitor	Phase Ib	106 patients Advanced/metastatic disease Most heavily pretreated 65.1% prior immunotherapy	Melanoma (35.8%) Triple‐Negative Breast Cancer (21.7%) Other solid tumors and lymphomas	ORR: 10.4% overall Group A (weekly ADU‐S100): 13.4% Group B (monthly ADU‐S100): 5.1% Median duration of response: 11.5 months Disease control rate: 29.2%	97.2% experienced ≥ 1 AE Most common AE: pyrexia (21.7%), injection site pain (19.8%), diarrhea (11.3%), fatigue (8.5%) No treatment‐related deaths	Meric‐Bernstam, Sweis, Kasper, et al. (2023)
E7766	Macrocycle‐bridged CDN STING agonist Kim et al. (2021)	Phase I	24 patients with advanced, refractory, or metastatic solid tumors; ECOG 0–1; all had injectable lesions	Heterogeneous solid tumors, including gastro‐esophageal, cutaneous, and visceral lesions	Pharmacodynamic activation of IFN pathway genes (IFN‐α, IFN‐β, IP‐10, IL‐6, TNF‐α, CD8, PD‐L1) Tumor stabilization in 8/24 patients (33.3%) One patient showed systemic tumor shrinkage despite low dose (75 μg) PD‐L1 and CD8 expression increased posttreatment Target engagement shown in injected and non‐injected lesions	Chills (50%–85.7%), Fever (40%–85.7%), Fatigue (30%–35.7%)	Luke, Pinato, et al. (2025)
MK‐2118	Non‐CDN STING agonist	Phase I	64 participants	Various (melanoma, breast, CRC, etc.)	No ORR 15% stable disease	Grade 3 AEs in 22% Common: injection site pain (52%), pyrexia (41%), anemia, fatigue	Luke, Sweis, et al. (2025)
MK‐2118^a^ + Pembrolizumab (IT)	Non‐CDN STING agonist + PD‐1 Inhibitor	Phase I	64 participants	Melanoma, mesothelioma, RCC, Cutaneous T‐Cell Lymphoma (CTCL)	ORR: 6% 1 CR, 3 PR 22% stable disease Increased IFNγ Increased IP‐10	Grade 3/4 AEs in 23% Common: pyrexia (42%), fatigue (33%), anemia, injection site reactions, cytokine release syndrome	Luke, Sweis, et al. (2025)
MK‐2118 + Pembrolizumab (sc)	Non‐CDN STING agonist + PD‐1 Inhibitor	Phase I	64 participants	Squamous cell carcinoma, acinic cell carcinoma, CTCL	ORR: 3.8% (1 CR, 1 PR) 23% stable disease Increased IFNγ and IL‐6 but no gene expression change	Grade 3/4 AEs in 11% Common: fatigue (41%), pneumonitis (1 Dose‐limiting toxicity)	Luke, Sweis, et al. (2025)
BI 1387446	Non‐CDN STING agonist	Phase I	41 patients	Malignant Solid Tumor	Stable disease in 46.2% of patients	Injection pain, fatigue, pyrexia, asthenia, hyperthyroidism; 1 Dose‐limiting toxicity (grade 3 fatigue and myalgia)	Calvo et al. (2023)
BI 1387446 + Ezabenlimab	Non‐CDN STING agonist + PD‐1 Inhibitor	Phase I	41 patients	Malignant Solid Tumor	Stable disease in 53.3% of patients	Injection site pain, fatigue, pyrexia, asthenia, hyperthyroidism	Calvo et al. (2023)
Ulevostinag	CDN STING agonist	Phase I	156 patients with advanced/metastatic solid tumors or lymphomas (± pembrolizumab)	HNSCC, TNBC, other solid tumors	Elevated IL‐6, IP‐10, IFN‐γ	Pyrexia 70%	Harrington et al. (2025)
Ulevostinag + Pembrolizumab	CDN STING agonist + PD‐1 inhibitor	Phase II	18 patients with previously untreated metastatic or unresectable, recurrent HNSCC	HNSCC	4/8 patients in combination group showed CR or PR Showed significant tumor shrinkage	Pyrexia (5 patients)	Harrington et al. (2025)

Abbreviations: AEs, adverse events; CR, complete response; ECOG, eastern cooperative oncology group performance status; HNSCC, head and neck squamous cell carcinoma; IFN‐γ, interferon‐gamma; NSCLC, non‐small cell lung cancer; ORR, objective response rate; PR, partial response; RCC, renal cell carcinoma; triple‐negative breast cancer.

^a^ Among the listed compounds, **MK‐2118** represents the only non‐CDN STING agonist to have demonstrated preliminary clinical efficacy to date.

## 7 New Derivatives in the Development of STING Agonists

The development of new STING agonists is driven by the limitations of existing compounds, including suboptimal potency, limited metabolic stability, poor bioavailability, and restricted immune activation. This section highlights recently investigated derivatives designed to overcome these challenges and improve therapeutic potential. Examined compounds include benzimidazole and benzamide derivatives, benzothiophene derivatives, 7‐deazapurine cyclic dinucleotide analogues, *S*‐acylthioalkyl ester‐based deoxyribose cyclic dinucleotide (SATE‐dCDN) derivatives, diphenyl derivatives, thieno[2,3‐*d*]imidazole derivatives, purine derivatives, and acridone derivatives. These derivatives were examined because they contain structurally similar or identical bioisosteric motifs present in existing STING agonists while offering the potential to enhance potency, selectivity, and stability. Presenting these new derivatives provides a rationale for their design and emphasizes SAR that may guide the development of more effective STING‐targeted therapies.

### 7.1 Benzimidazole and Benzamide Derivatives

**FIGURE 5:**
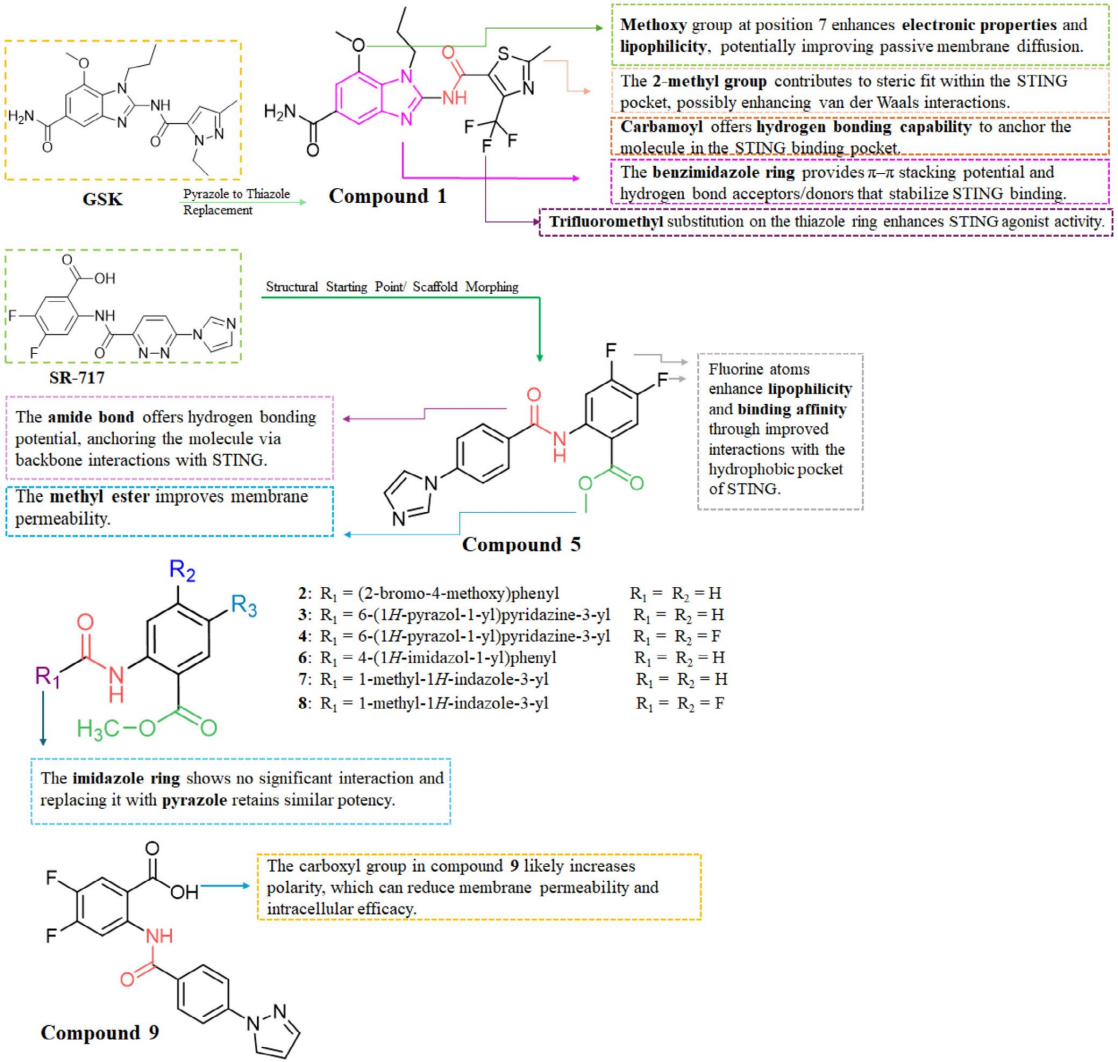
The lead compounds **GSK** and **SR‐717** served as structural starting points for scaffold morphing and bioisosteric replacements to improve metabolic stability and potency in compounds **1–9**.

A series of benzimidazole‐based STING agonists was designed to address the pharmacological limitations of reference molecules, specifically **SR‐717** and **GSK** [2‐(1‐ethyl‐3‐methyl‐1*H*‐pyrazole‐5‐carboxamido)‐7‐methoxy‐1‐propyl‐1*H*‐benzo[*d*]imidazole‐5‐carboxamide], a potent agonist previously reported by Ramanjulu et al. (2019). As illustrated in the structural evolution scheme (Figure 5), the **GSK** molecule served as the primary structural starting point for the design of the benzimidazole series. While the **GSK** reference exhibited moderate potency, it was hindered by a very short plasma half‐life, necessitating structural optimization. To address this, the central pyrazole ring of the **GSK** molecule was replaced with a thiazole core to design compound **1**. Additionally, a trifluoromethyl group was incorporated onto the thiazole ring to effectively block oxidative metabolism sites, thereby improving the compound's effective intracellular concentration. Consequently, compound **1** emerged as a standout candidate, inducing IFN‐β secretion levels in human THP‐1 cells that significantly exceeded those of the **GSK** reference. Pharmacokinetic evaluation in rats confirmed that compound **1** achieved a marked improvement in plasma half‐life and oral bioavailability compared to **GSK**, validating the design strategy. Among the active derivatives, compound **5**, designed by replacing the pyridazine ring of the reference molecule **SR‐717** with a pyrimidine core, demonstrated robust IFN‐β secretion, notably outperforming the reference molecule. The primary structural deviations from **SR‐717** involve the incorporation of an ester moiety and a pyrazole ring in compound **4**. For compound **6**, the modifications consist of the addition of an ester moiety coupled with the absence of fluorine atoms on the phenyl ring. A key distinction observed was species specificity: Compound **5** successfully activated mSTING in RAW 264.7 cells, whereas compound **1** and the reference molecules remained inactive in this system. Comparisons revealed that the ester analogue, compound **5**, was superior to compound **9**. The reduced potency of compound **9**, a distinct carboxylic acid analogue, was attributed to the increased polarity of the free acid moiety, which likely impairs membrane permeability. Molecular docking studies corroborated the experimental findings, showing that both compounds **1** and **5** form strong hydrogen bonds with critical hSTING residues such as Arg238, mimicking the binding mode of **SR‐717**. Western blot analysis further confirmed pathway activation through the phosphorylation of STING, TBK1, and IRF3. Overall, compounds **1** and **5** were identified as highly promising agonists, combining potent in vitro activity with favorable pharmacokinetic properties derived from the systematic optimization of reference scaffolds (Fan et al. 2025).

### 7.2 Amidobenzimidazole Derivatives

**FIGURE 6:**
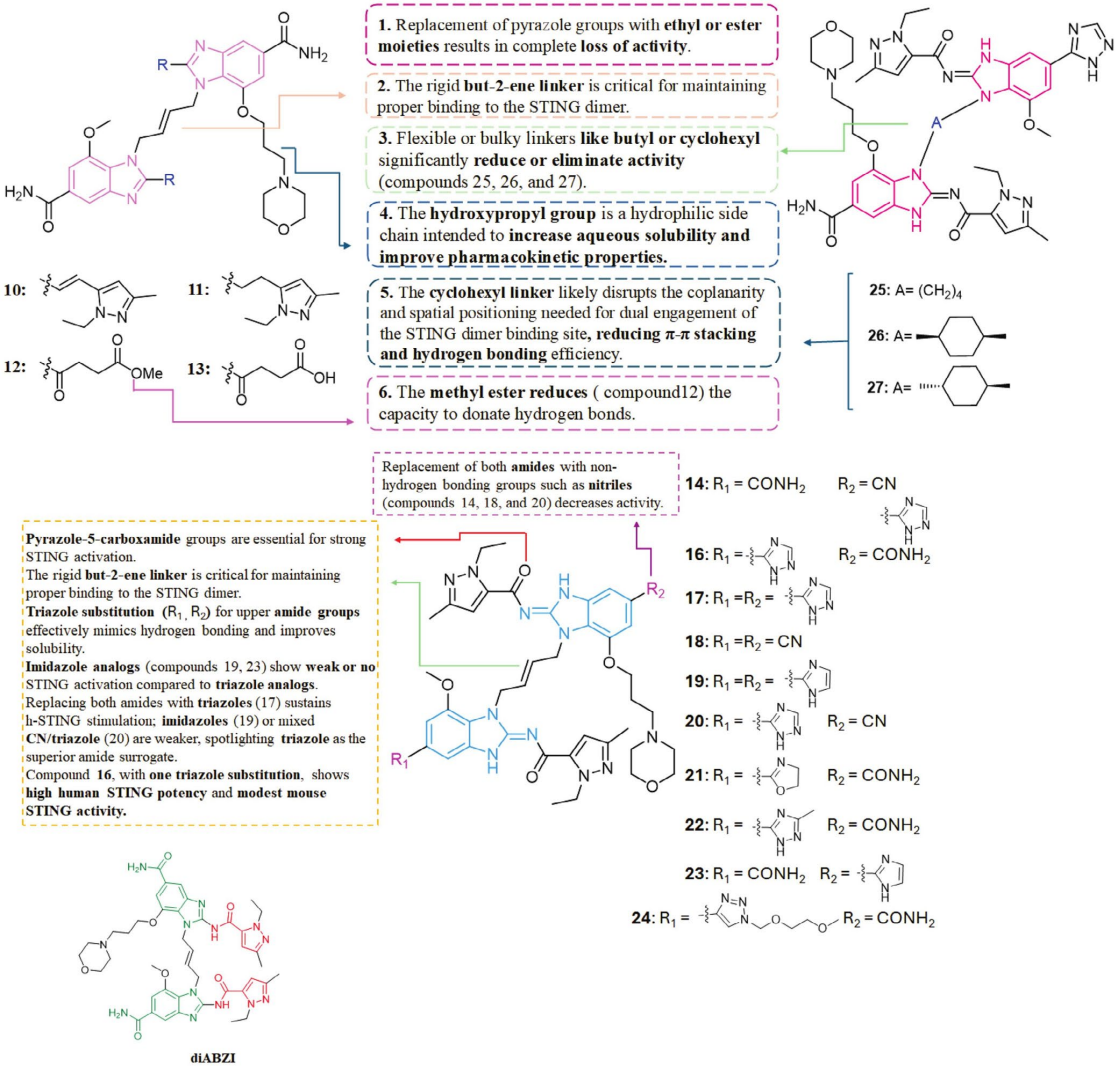
SAR analysis of amidobenzimidazole‐containing molecules.

A novel series of amidobenzimidazole‐based STING agonists was designed and synthesized to evaluate their therapeutic potential (Figure 6). Compound **diABZI** is the most potent STING agonist identified, with an EC_50_ < 0.0001 μM for hSTING and 3.0 μM for mSTING, due to its symmetric structure featuring two pyrazole‐5‐carboxamide groups and two benzo[*d*]imidazole‐5‐carboxamide moieties. SAR studies highlighted the critical role of the pyrazole rings. Specifically, these structural alterations were divided into two distinct groups: pyrazole modifications (compounds **10** and **11**) and the complete removal of the pyrazole core (compounds **12** and **13**). Both approaches resulted in a significant loss of activity. Conversely, the upper amide groups demonstrated greater structural plasticity. Triazole substitutions (compound **17** and **16**) showed strong activity on hSTING (e.g., compound **16**: EC_50_ = 0.24 μM) and modest mSTING activity (39.51 μM), whereas imidazole (compound **19**) and cyano groups (compound **18**) were less effective. Among all tested molecules, compound **16** showed the best balance: high potency for hSTING, modest activity for mSTING, > 20‐fold improved aqueous solubility (71.33 mg/L vs. 3.23 mg/L for **diABZI**), and better tolerability in vivo. On the other hand, compounds in which the amide groups were replaced with non–hydrogen‐bonding functionalities such as nitriles (e.g., **18** and **20**), or in which the essential pyrazole moiety was removed (**12**), exhibited weak hSTING activation (EC_50_ values of > 50, 26.0, and 29.1 μM, respectively) and were completely inactive against mSTING (EC_50_ > 50 μM). In contrast, compounds such as imidazole compound **19**, dihydrooxazole compound **21**, and methyl triazole compound **22** exhibited moderate activity against hSTING, with EC_50_ values of 3.28, 1.54, and 2.55 μM, respectively, while remaining inactive on mSTING. Interestingly, triazole compound **16** showed strong activation of hSTING, achieving an EC_50_ of 0.24 μM, which was only slightly less potent than the reference **diABZI** (EC_50_ = 0.13 μM). Modifying the central but‐2‐ene linker or introducing bulky solubilizing groups often reduced activity (e.g., compounds **25–27**), emphasizing the importance of rigid geometry and careful polarity management in drug design. In the crystal complex (PDB: 6DXL), compound **16** shares key interactions with the reference molecule (Ramanjulu et al. 2019). Its triazole replaces the ligand's amido group, forming a hydrogen bond with Ser241 and a π‐π interaction with Tyr240, while the pyrazole nitrogen forms a new hydrogen bond with Ser162. The right‐side 3‐morpholinopropoxy chain is oriented vertically and does not interact with hSTING (Song et al. 2021).

### 7.3 Thieno[2,3‐*d*]Imidazole Derivatives

**FIGURE 7:**
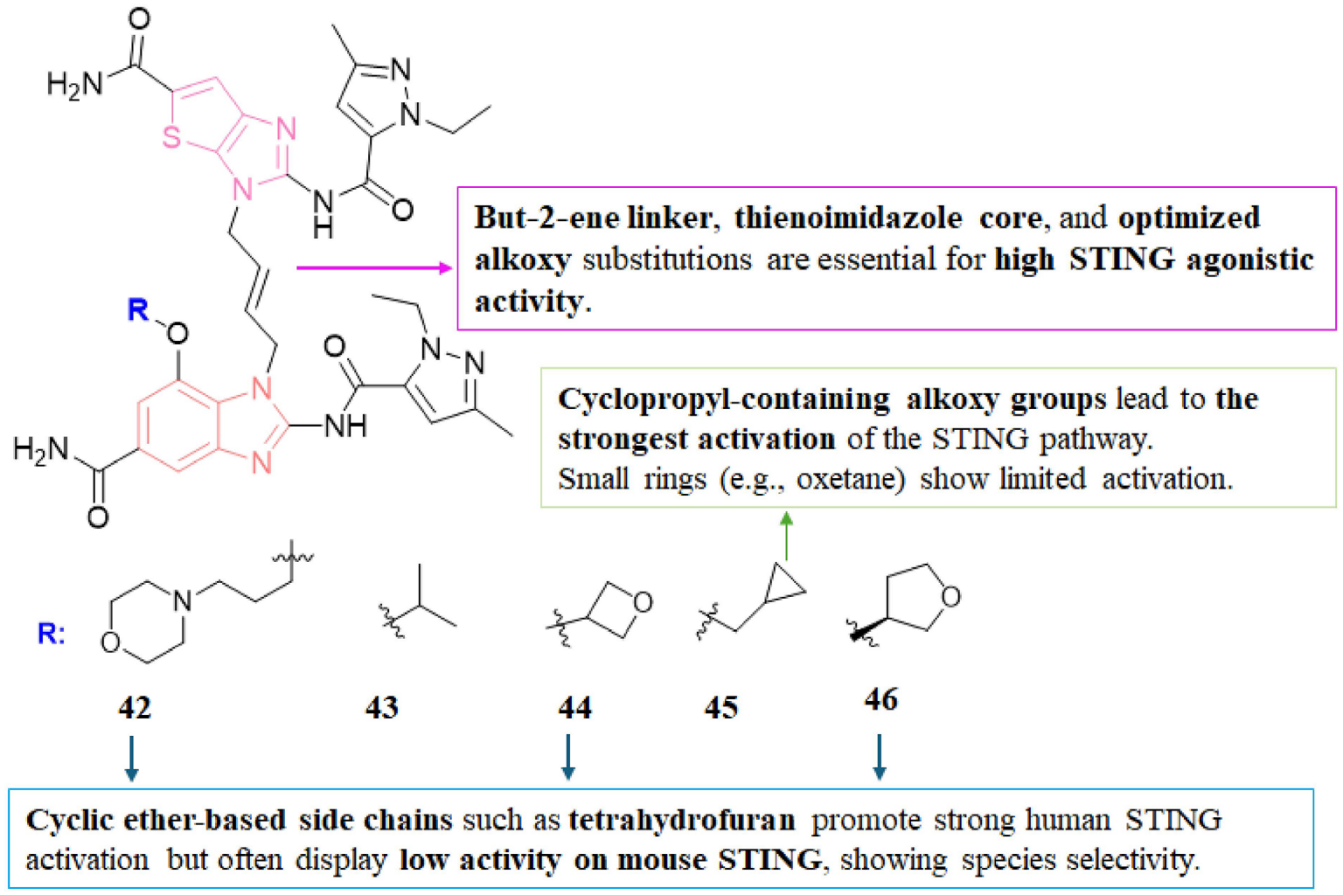
Molecular structure analysis of thieno[2,3‐*d*]imidazole derivatives.

A novel series of thieno[2,3‐*d*]imidazole derivatives were designed as STING agonists, among which compound **42** emerged as a highly potent and selective candidate with significant antitumor activity and promising SAR insights (Figure 7). The carboxamide group is essential for STING activation, likely due to its ability to form hydrogen bonds with key Serine residues. Modifying this group significantly reduces or abolishes activity. Compound **42** demonstrated an EC_50_ of 1.2 nM against hSTING and 32.82 μM against mSTING, confirming its species selectivity and high potency for hSTING. Structural optimization showed that the but‐2‐ene linker dramatically enhanced activity, as seen in compound **45**. Moreover, analogs **43–46** confirmed that alkoxy substituents contributed to hSTING potency, though none surpassed compound **42**. Compound **45**, bearing a cyclopropylmethoxy group, showed the highest ISG activation for hSTING (141.02‐fold) and a moderate effect on mSTING (12.61‐fold), with an EC_50_ of 0.28 μM (hSTING). Compound **46**, which featured a tetrahydrofuran‐3‐yloxy substituent, displayed strong hSTING activation (80.35‐fold) but very weak mSTING activity (1.50‐fold), indicating species selectivity. Compounds **43** (isopropoxy) and **44** (oxetane ring) also activated hSTING with respectable fold changes (60.37 and 49.24, respectively), but showed moderate mSTING activity and weaker EC_50_s compared to compound **42**. Pharmacokinetically, compound **42** showed improved parameters over the reference compound **diABZI**, including a longer half‐life (*T*_1/2_ = 1.62 h) and greater volume of distribution (*V*_ss_ = 1516 mL/kg) after IV dosing, indicating favorable systemic exposure. Functionally, **42** activated both ISG and NF‐κB pathways in THP1‐Dual cells in a STING‐dependent manner, confirmed by complete loss of activity in STING knockout cells. It induced phosphorylation of TBK1 and IRF3, and significantly increased IFN‐β and IP‐10 production, further confirming downstream STING activation. Importantly, compound **42** was active across multiple hSTING isoforms (H232 and R232) and in mouse macrophage cells (Raw‐Lucia), indicating broad cellular efficacy. In in vivo tumor models, compound **42** showed exceptional efficacy. In the CT26 colon tumor model, IV administration at 10 mg/kg led to complete tumor regression in 6 of 9 mice, while IT administration at 5 mg/kg cured 8 of 10 mice, all without observable weight loss or toxicity. Compound **42** demonstrated robust, dose‐dependent tumor growth inhibition in vivo without causing significant changes in body weight. Treatment resulted in a marked elevation of plasma IFN‐β and IP‐10 levels, confirming systemic immune activation. Compound **42** binds directly to multiple h‐STING isoforms with high potency. Differential scanning fluorimetry (DSF) assays demonstrated that it markedly increases the thermal stability of all tested isoforms, while homogeneous time‐resolved fluorescence (HTRF) analysis determined an IC_50_ of 0.60 nM for human wild‐type STING (R232). These findings support that compound **42** effectively stimulates antitumor immunity via STING pathway activation, combining high potency, favorable pharmacokinetics, broad isoform activity, and strong in vivo efficacy, making it a preclinical candidate of considerable promise for cancer immunotherapy (Niu et al. 2022).

### 7.4 Benzothiophene Derivatives

**FIGURE 8:**
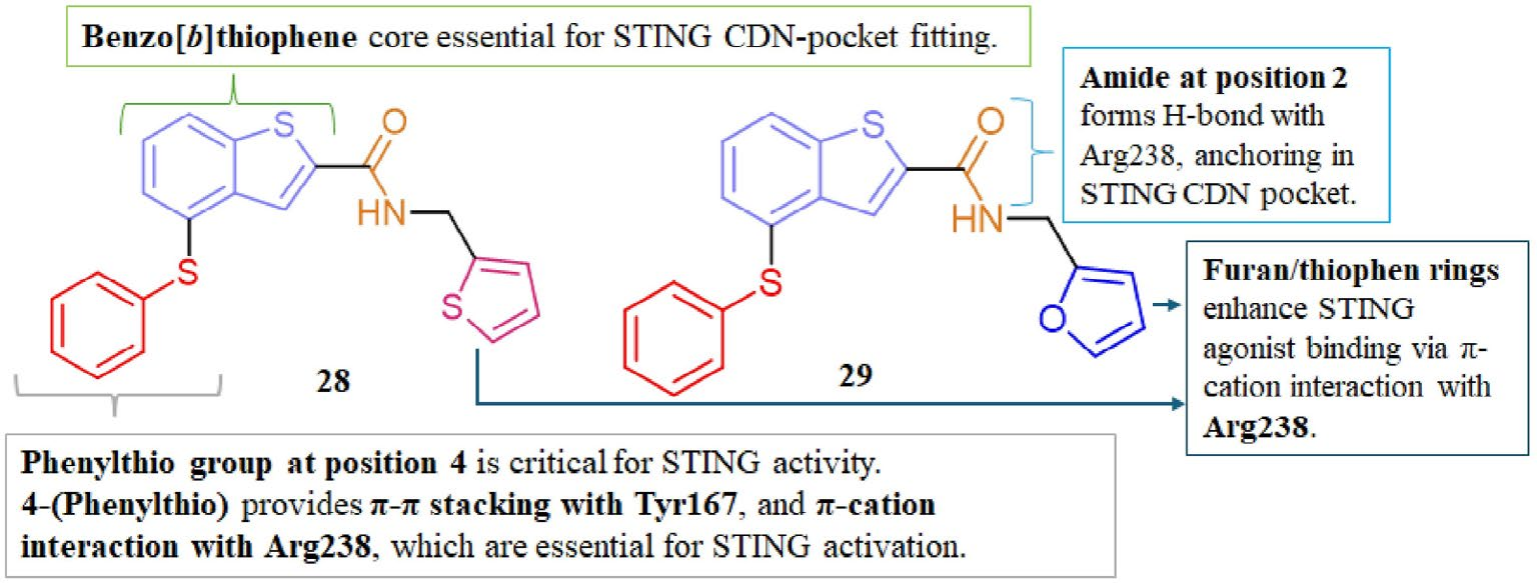
SAR analysis of benzothiophene‐containing molecules.

A series of benzo[*b*]thiophene‐2‐carboxamide derivatives were synthesized to evaluate their STING agonistic activity, with compounds **28** and **29** emerging as marginally active candidates (Figure 8). When tested in human THP1‐Dual cells at a concentration of 50 μM, both compounds induced more than a twofold increase in hSTING activation compared to baseline, although their potency remained considerably lower than the reference agonist **ADU‐S100**, which exhibits EC_50_ values in the micromolar range in these assays. Western blot analyses demonstrated that treatment with **28** and **29** led to increased phosphorylation of downstream signaling molecules TBK1 and IRF3, effects that were absent in STING‐knockout THP1‐Dual cells, confirming STING pathway specificity. Importantly, both compounds showed no cytotoxicity in THP1‐Dual cells up to 20 μM, supporting a favorable in vitro safety profile. Molecular docking using PDB structure 6CFF revealed that compounds **28** and **29** bind the CDN‐binding domain of hSTING in a “U‐shaped” conformation. This binding is stabilized by two canonical hydrogen bonds between the ligand's carbonyl group and Arg238, a π‐π stacking interaction with Tyr167, and a π‐cation interaction involving the thiophene/furan moiety and the guanidine side chain of Arg238. However, unlike the more potent STING agonist **MSA‐2**, compounds **28** and **29** lack hydrogen bond interactions with Ser162 and Thr263, which may partially account for their limited activity. Hydrogen bonds, π‐π stacking, and π‐cation interactions with residues such as Arg238, Tyr167/Tyr240, and Ser162/Ser241 are the most critical interactions for STING agonists. The presence of a phenylthiol group at the 4‐position of the benzo[*b*]thiophene ring was found to be critical for STING stimulation, as shown by the superior performance of these two analogs within the series. In conclusion, while **28** and **29** show only modest hSTING agonistic activity, their selective activation of STING, defined interaction profile, and low cytotoxicity make them promising lead structures for further optimization (Zhou et al. 2023).

**FIGURE 9:**
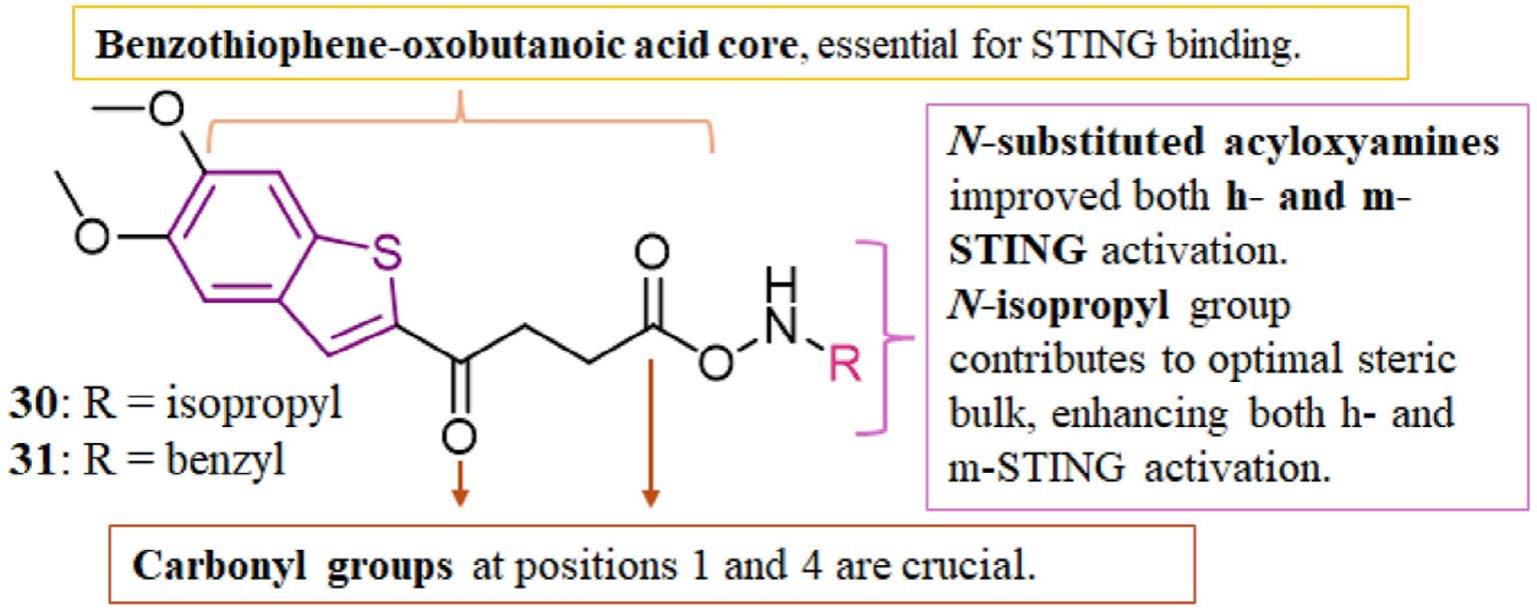
Chemical structure and SAR analysis of *N*‐substituted acyloxyamino derivatives.

A series of *N*‐substituted acyloxyamino derivatives were developed based on the benzothiophene oxobutanoic acid scaffold of the well‐documented STING agonist **MSA‐2** (Figure 9). SAR analysis revealed that compounds **30** and **31** exhibited the most potent STING activation effects. The reference compound **MSA‐2** activated hSTING significantly; however, the newly designed analogues aimed to surpass this baseline efficacy. In contrast, compound **30** demonstrated superior activity with 25.4‐fold activation of hSTING (EC_50_: 2.55 μM) and 19.3‐fold activation of mSTING, surpassing both **MSA‐2** and the clinical‐stage STING agonist **ADU‐S100**, which has an hSTING EC_50_ of 5.34 μM. Compound **31** showed the strongest activity on mSTING, with a 25.3‐fold activation and 22.4‐fold activation on hSTING (EC_50_: 6.14 μM). Cytokine secretion assays confirmed that compound **30** induced the highest levels of IFN‐β and IP‐10 in THP1‐Dual cells. Further mechanistic studies, including experiments using STING‐knockout cells and TBK1 inhibition, demonstrated that compound **30** specifically activates the STING pathway. DSF confirmed the direct binding of compound **30** to both human and mSTING proteins, stabilizing their structure. In vivo, compound **30** significantly inhibited tumor growth in the low‐immunogenicity B16F10 melanoma model after both IT and oral administration, without observable toxicity or body weight loss. Remarkably, IT treatment with compound **30** in the CT26 colon cancer model led to complete tumor eradication and prevention of tumor re‐challenge, indicating induction of immune memory. Docking was conducted based on the hSTING structure (PDB ID: 6UKM). The ketone group of compound **30** forms a hydrogen bond with the guanidinium side chain of Arg238. The acyl group of the acyloxyamine moiety forms a hydrogen bond with Thr263. The acyloxyamino group interacts with two water molecules via hydrogen bonds at favorable angles, which likely contributes to stabilizing the STING protein. The interaction with Arg238 is particularly important for agonist activity. Overall, compound **30** stands out as the best STING agonist among the tested compounds, offering stronger dual human and mSTING activation, more potent cytokine induction, and superior in vivo antitumor efficacy compared to **MSA‐2** and **ADU‐S100** (Shen et al. 2022).

### 7.5 7‐Deazapurine Cyclic Dinucleotide Analogues

**FIGURE 10:**
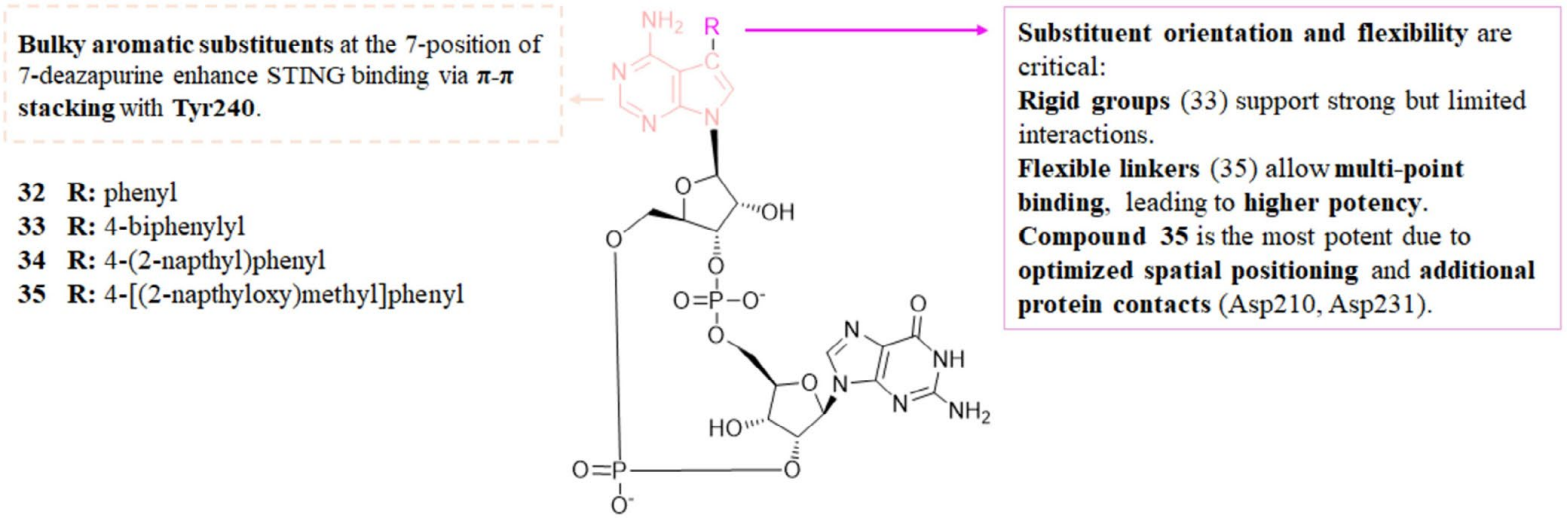
Chemical structure and SAR analysis of compounds **32–35**.

The synthesized 7‐substituted 7‐deazapurine CDNs represent a novel class of STING agonists exhibiting enhanced activity compared to the natural ligand 2′3′‐cGAMP (Figure 10). Most of the interactions observed for compounds **32** (PDB ID: 8A2H), **33** (PDB ID: 8A2J), **34** (PDB ID: 8A2I), and **35** (PDB ID: 8A2K) occur within the STING ligand‐binding domain. These compounds feature large aromatic substituents at the 7‐deazaadenosine position, enabling unique π‐π stacking interactions with Tyr240 within the STING binding pocket, which contribute to increased ligand stability. Structural studies revealed that these bulky substituents induce a shift in the AMP moiety positioning, displacing Arg238 and allowing new interactions not observed with natural CDNs. Notably, compound **35**, bearing a flexible methoxy‐linked naphthyl group, demonstrated additional T‐shaped π‐stacking with Tyr240 and π‐amide/anion interactions with Asp231 and Asp210, resulting in superior STING activation potency. π‐π stacking interactions between the aromatic substituents and Tyr240 play a key role in stabilizing CDN‐STING complexes. Biochemical assays confirmed that compounds **32** and **35** elicited broad cytokine responses, including the induction of IFNγ, TNFα, and IFNα. Although the potency differences across the series were relatively minor, compounds **32 *and* 35** consistently exhibited the highest activity, effectively outperforming analogues **33** and **34**. Consequently, these two molecules emerged as the most promising lead candidates within this series of modified CDNs (Vavřina et al. 2022).

### 7.6 *S*‐Acylthioalkyl Ester Based Deoxyribose Cyclic Dinucleotide (SATE‐dCDN) Derivatives

**FIGURE 11:**
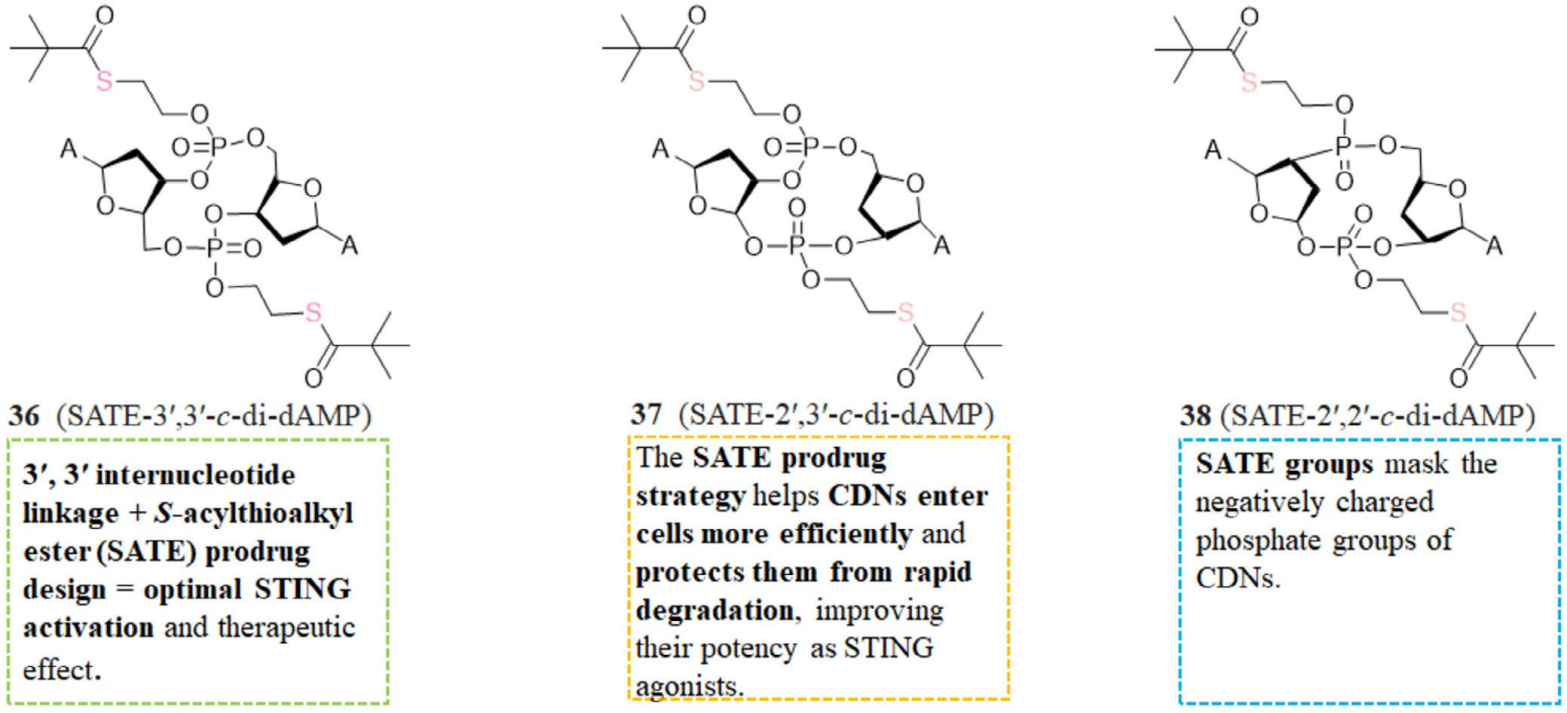
SAR analysis of SATE‐dCDN derivatives.

Three *S*‐acylthioalkyl ester (SATE) based prodrugs of deoxyribose cyclic dinucleotides (SATE‐dCDNs), identified as compounds **36** with 3′,3′ linkage, **37** with 2′,3′ linkage, and **38** with 2′,2′ linkage, were developed to enhance STING activation by improving cell penetration, serum stability, and overall bioactivity in comparison to natural CDNs and **ADU‐S100**. Within this series, compound **36** emerged as the most potent STING agonist, exhibiting the lowest EC_50_ values across all transfection conditions and achieving an exceptionally high maximum activation (Figure 11). Compound **37** also demonstrated strong agonistic activity, although it remained slightly less potent than Compound **36**. In contrast, compound **38** showed substantially reduced potency, with activity levels orders of magnitude lower than the lead candidates. Notably, Compound 36 displayed significantly superior potency compared to standard references such as **2′,3′‐cGAMP**, **3′,3′‐c‐di‐dAMP**, and **ADU‐S100**, underscoring its potential as a highly effective STING activator. Even in the presence of transfection agents like lipofectamine or digitonin, compound **36** maintained superior activity, suggesting that it has excellent cell membrane permeability and efficiently releases active dCDNs inside cells. The maximum fold induction of STING pathway activation by compound **36** was measured as 4.44 times that of 2′,3′‐cGAMP. Compounds **36–38** were evaluated for serum stability in 20% FBS. Compound **36** exhibited the longest half‐life (72 h) compared to **37** (29 h) and **38** (41 h), indicating that the 3′,3′ internucleotide linkage enhances stability. The parent dCDN (3′,3′‐c‐di‐dAMP) remained intact at 72 h, whereas 80% of 2′,3′‐cGAMP degraded. Overall, compound **36** demonstrated superior serum stability relative to natural 2′,3′‐cGAMP and **ADU‐S100**, highlighting their potential for prolonged in vivo activity. In a CT26‐Luc tumor‐bearing mouse model, IT injection of compound **36** led to complete tumor regression in all mice by day 9 and significantly increased the expression of immune‐related cytokines such as interferon beta and interleukin‐6. Specifically, the prodrug treatment triggered a massive induction of type I interferons compared to the control. Additionally, mice treated with compound **36** survived beyond 60 days and showed only minimal, transient weight loss, indicating both strong efficacy and low toxicity. Compared to **ADU‐S100**, which resulted in a 75% survival rate at day 50, compound **36** demonstrated greater immunostimulatory and antitumor effects. These results indicate that the combination of a deoxyribose backbone, SATE prodrug design, and 3′,3′ internucleotide linkage in compound **36** provides clear structural advantages, making it a highly promising candidate for STING‐targeted cancer immunotherapy (Xie et al. 2022). Although SATE‐dCDN derivatives are promising prodrugs that may improve the cellular delivery and in vivo half‐life of CDNs, data on their safety profile in humans remain limited.

### 7.7 Diphenyl Derivatives

**FIGURE 12:**
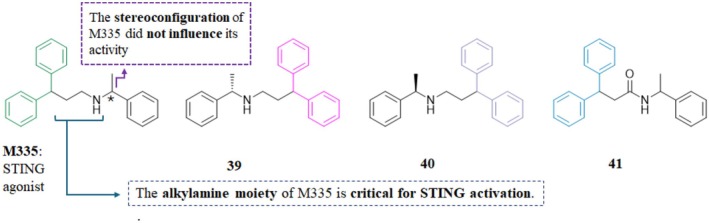
Analysis of chemical structure and SAR of diphenyl derivatives.

The novel small‐molecule STING agonist **M335** demonstrates potent antitumor activity by activating the STING‐dependent TBK1‐IRF3‐IFN signaling pathway, thereby enhancing innate immune responses. In multiple tumor models, including MC38, CT26, and B16F10, M335 significantly suppressed tumor growth without inducing toxicity or weight loss, confirming its favorable safety profile. Notably, **M335** did not trigger apoptosis at therapeutic concentrations, indicating that its antitumor efficacy is immune‐mediated rather than cytotoxic. It promoted substantial infiltration of CD8^+^ T cells and NK cells into tumors and spleens and increased serum levels of proinflammatory cytokines such as IL‐6, underscoring its capacity to stimulate immune activation. **M335** was effective through both IT and intraperitoneal administration, offering flexibility in therapeutic delivery. SAR studies revealed that the alkylamine moiety is indispensable for STING activation, while stereochemistry had no effect: both optically pure enantiomers, **39** (*R*‐form) and **40** (*S*‐form), showed comparable biological activity, confirming that the configuration does not influence efficacy. Additionally, a related compound, **41**, which lacked the alkylamine group, exhibited markedly reduced activity, further validating the critical role of this moiety (Figure 12). Although **M335** displayed only modest binding affinity in surface plasmon resonance (SPR) and thermal shift assays, it effectively induced STING oligomerization, ER‐to‐Golgi translocation, and phosphorylation of downstream targets such as TBK1 and IRF3. DMET prediction for **M335** indicated a low solubility level (2) and very high blood–brain barrier (BBB) penetration (level 0, brain‐to‐blood ratio > 5:1). The compound was predicted to inhibit CYP2D6 and to be hepatotoxic, while showing good absorption (level 0) and high plasma protein binding (PPB). Toxicity predictions suggested that **M335** is a noncarcinogen in both female and male mice and rats, non‐mutagenic in the Ames test, and exhibits no developmental toxicity potential. Additionally, it showed only mild skin irritancy, and the overall weight of evidence classified it as noncarcinogenic. Pharmacokinetic and toxicity predictions supported **M335**'s druggability, showing good oral absorption, blood–brain barrier permeability, and low toxicity risks. In conclusion, **M335**, along with **39** and **40**, represents a promising new class of STING agonists with a distinct activation mechanism and significant antitumor potential through immune modulation (Zhao et al. 2024).

### 7.8 Purine Derivatives

**FIGURE 13:**
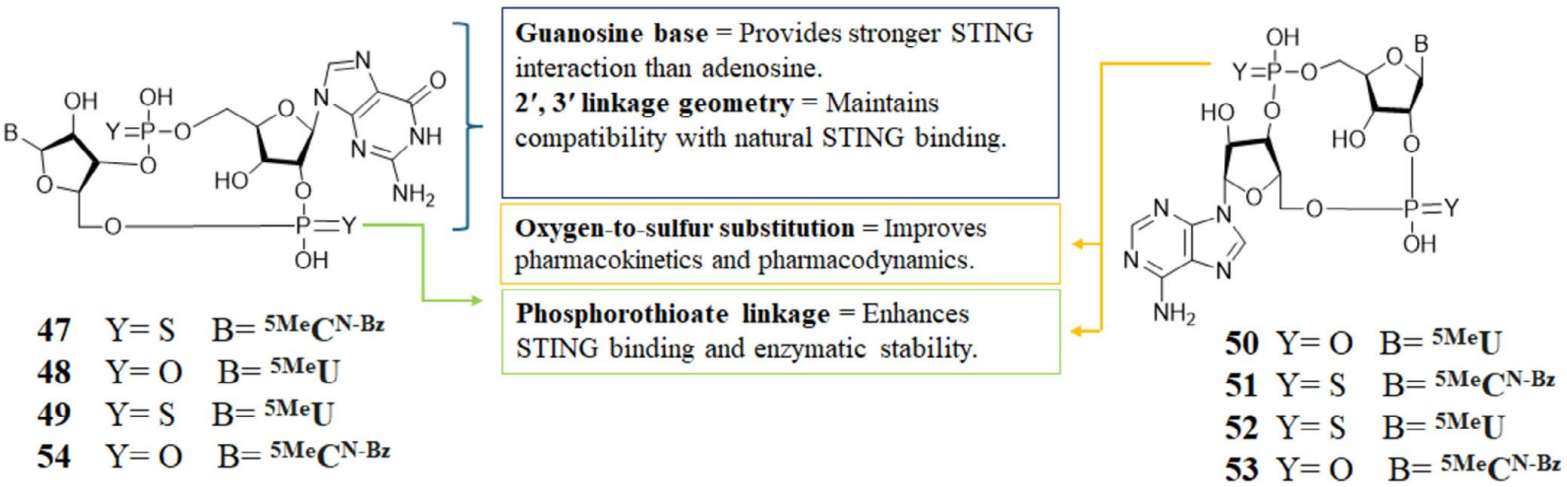
Structural overview of synthetic CDN derivatives and their structure–activity relationship.

Synthetic CDNs with phosphorothioate and phosphodiester internucleotide linkages were developed to enhance bioactivity, binding affinity, and enzymatic stability. Among these, compound **47** emerged as the most potent molecule (X. Xie et al. 2020). A SPR assay was established to quantify the binding affinity of each compound to hSTING, using minimal sample volume and without requiring labeling or pretreatment. The *K*_D_ of compound **47** was measured as 0.038 μM, significantly lower than that of natural cGAMP (*K*_D_ = 0.543 μM), indicating a much stronger interaction with hSTING. This superior binding was further attributed to the presence of guanosine moieties and phosphorothioate linkages in compound **47**, which outperformed analogs with adenosine bases and standard phosphodiester bonds. For comparison, compound **48** (phosphodiester, guanosine) had a *K*_D_ of 7.581 μM, while compound **49** (phosphorothioate, guanosine) improved to 0.599 μM. Similarly, compound **50** (phosphodiester, adenosine) showed poor affinity (*K*_D_ = 11.704 μM), while compound **51** (phosphorothioate, adenosine) improved to 0.728 μM. Cytokine induction assays in human THP‐1 cells demonstrated that the phosphorothioate CDNs (**47**, **49**, **51**, **52**) led to significantly greater IFN‐β expression compared to their phosphodiester counterparts. Compound **47**, in particular, showed the strongest ability to induce IFN‐β, CXCL10, and IL‐6 mRNA levels, exceeding those induced by cGAMP. This supports the conclusion that sulfur substitution at the phosphodiester linkage increases both binding affinity and cellular activity, making phosphorothioate modification a critical SAR determinant for potent STING agonism. Enzymatic stability studies demonstrated that CDNs containing phosphodiester linkages (e.g., compounds **48**, **50**, **53**, and **54**) were rapidly degraded by serum enzymes and nuclease P1, exhibiting half‐lives of 2–4 min in serum and 7–10 min with nuclease P1. In contrast, phosphorothioate analogs like compound **47** remained largely intact after 2 h of incubation, confirming their enhanced resistance to enzymatic degradation. Molecular docking simulations further supported these findings. Compound **47** exhibited a more favorable docking score with hSTING (−11.83 kcal/mol) compared to cGAMP (−9.76 kcal/mol) and **54** (−10.88 kcal/mol). The enhanced binding of compound **47** was attributed to several key interactions: hydrogen bonding between the sulfur atom in the phosphorothioate group and key amino acid residues (Gly166 and Ser162), π‐π stacking, and van der Waals interactions with the STING binding pocket (PDB: 4LOH). The incorporation of phosphorothioate linkages significantly enhanced both binding affinity to hSTING and enzymatic stability compared to traditional phosphodiester bonds. Guanosine‐containing analogs exhibited stronger interactions with STING than those containing adenosine, indicating that base identity influences binding efficacy. In addition, maintaining a 2′,3′ linkage geometry, as found in natural cGAMP, proved effective when combined with chemical modifications that increase molecular stability. Replacing oxygen atoms with sulfur at the phosphate linkage improved molecular interactions with STING and also enhanced the pharmacokinetic and pharmacodynamic properties of the compounds. Compound **47**, which incorporates guanosine bases and phosphorothioate linkages in a 2′, 3′ cyclic configuration, demonstrated superior binding affinity to STING, greater cytokine induction, and improved stability compared to cGAMP and other analogs. The key residue Ser162 plays a critical role in stabilizing the ligand‐protein complex (Figure 13).

### 7.9 Acridone Derivatives

**FIGURE 14:**
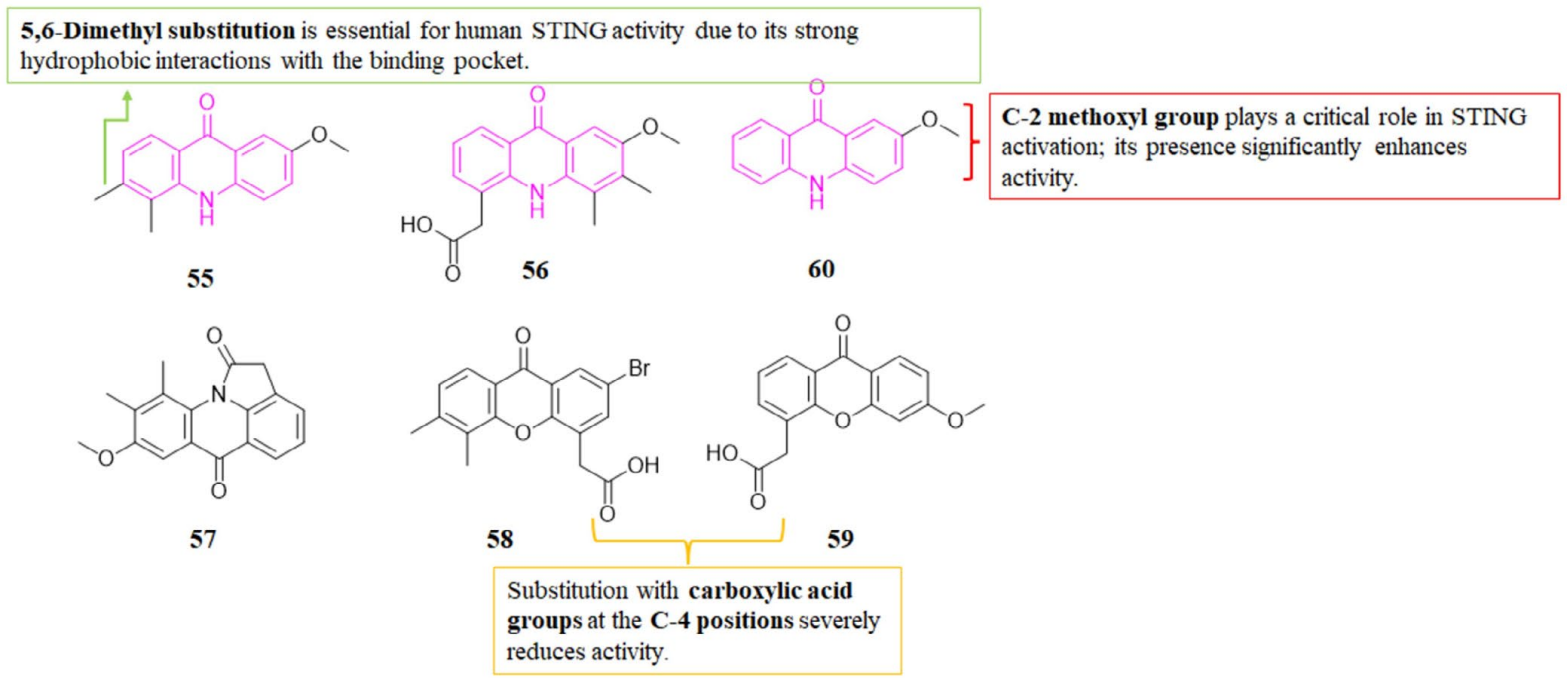
Chemical structures and SAR analysis of the a‐series acridone‐based molecules.

A series of acridone‐based small molecules were synthesized and screened as potential STING agonists (Hou et al. 2020). Compound **55** emerged as the lead candidate, showing robust dual‐species activation for both hSTING and mSTING. It achieved a maximal response (*E*_max_) exceeding that of the control, with an efficacy profile mirroring or surpassing the natural ligand **2′3′‐cGAMP**. In contrast, established murine‐selective agonist **DMXAA** failed to activate hSTING, serving as valid negative controls in human cell lines. Other analogues, specifically compounds **56** and **57**, demonstrated cross‐species activity but with significantly reduced potency compared to the lead compound **55**. These derivatives, characterized by C7‐methoxyl substitutions, exhibited only moderate efficacy, suggesting that this specific modification provides suboptimal functional benefits. Conversely, compounds **58**, **59**, and **60** remained murine‐specific with weak activity, showing no detectable response in hSTING‐expressing cells. SAR analysis highlighted two critical design features: the 5,6‐dimethyl substitutions were identified as essential for hSTING activation, likely facilitating hydrophobic interactions within the binding pocket, while the C‐2 methoxyl group in compound **55** emerged as a key determinant of potency, as its removal or modification significantly compromised function. Binding affinity assays further confirmed that compound **55** formed the most stable ligand‐STING complex among the synthetic series, distinguishing it from nonbinding controls like **DMXAA**. These attributes position compound **55** as a promising candidate for further non‐CDN agonist development (Figure 14).

## 8 Conclusion

The cGAS‐STING pathway is a pivotal regulator of antitumor immunity, linking innate and adaptive responses. Detection of cytosolic dsDNA triggers cGAMP production, activating STING and inducing type I interferons and proinflammatory cytokines. This enhances dendritic cell function, cytotoxic T lymphocyte activity, and NK cell responses, making STING an attractive target for cancer immunotherapy. Pharmacological STING agonists are classified into CDN analogs and non‐CDN small molecules. While CDNs, such as **ADU‐S100** and **Ulevostinag**, closely mimic natural ligands, they often suffer from poor cellular uptake, rapid enzymatic degradation, and limited systemic bioavailability. Non‐CDN agonists, including **diABZI**, **MSA‐2**, and newer derivatives, overcome many of these limitations by improving membrane permeability, pharmacokinetics, tumor‐selective activation, and systemic delivery. SAR analyses indicate that potent STING activation requires rigid, symmetric scaffolds for π‐π stacking, key hydrogen‐bond interactions (Arg238, Ser162, Glu260, Thr263), and electron‐withdrawing groups for stability, while highly polar groups can impair membrane permeability and activity. Recent investigations have identified several promising classes of derivatives. Benzimidazole derivatives, exemplified by compounds **1** and **5**, demonstrate potent STING activation in human cells with enhanced IFN‐β secretion, where structural features such as the trifluoromethyl‐substituted thiazole ring and Arg238‐mediated hydrogen bonding are critical for their activity. Amidobenzimidazole derivatives, exemplified by **diABZI** and **compound 16**, display high potency and balanced activity for mSTING, with the pyrazole‐5‐carboxamide core being critical for function. Thieno[2,3‐*d*]imidazole derivatives, such as compound **42**, display highly potent and selective hSTING activation, with the carboxamide group, but‐2‐ene linker, and alkoxy substituents being critical for activity, while showing robust IFN‐β induction and strong in vivo antitumor effects. Benzothiophene derivatives (compounds **28, 29, 30, 31**) and SATE‐dCDN prodrugs (compound **36**) improve cellular uptake, serum stability, and dual human/mSTING activation. Diphenyl derivatives (**M335**) stimulate immune‐mediated antitumor responses via the TBK1‐IRF3‐IFN pathway without cytotoxicity, while purine derivatives (compound **47**) leverage phosphorothioate modifications for enhanced binding affinity and enzymatic resistance. Acridone derivatives (compound **55**) demonstrate potent dual‐species STING activation, with 5,6‐dimethyl and C2‐methoxyl substitutions being critical for receptor binding and cytokine induction.

In summary, these studies underscore that next‐generation STING agonists should combine rigid, symmetric scaffolds, π‐π stacking aromatic motifs, critical hydrogen‐bonding groups, electron‐withdrawing substituents, and optimized linkers, exhibiting EC_50_ values that span from low nanomolar to micromolar scales. Prodrug strategies (e.g., SATE‐dCDNs) and careful modulation of polarity further enhance cellular uptake and systemic delivery. Rational integration of these design principles enables the development of potent, selective, and clinically translatable STING agonists, advancing the prospects of cancer immunotherapy.

## Author Contributions

**Dilay Kahvecioglu Cicek:** conceptualization, writing – original draft, supervision, resources, writing – review and editing.

## Funding

The author has nothing to report.

## Ethics Statement

The author has nothing to report.

## Conflicts of Interest

The author declares no conflicts of interest.

## Data Availability

No new data were generated during this study. All data analyzed during this review are included in this published article and its cited references.
